# Differential Gene Expression and Immune Cell Infiltration in Carotid Intraplaque Hemorrhage Identified Using Integrated Bioinformatics Analysis

**DOI:** 10.3389/fcvm.2022.818585

**Published:** 2022-05-17

**Authors:** Xiaoshuo Lv, Feng Wang, Mingsheng Sun, Congrui Sun, Xueqiang Fan, Bo Ma, Yuguang Yang, Zhidong Ye, Peng Liu, Jianyan Wen

**Affiliations:** ^1^Department of Cardiovascular Surgery, China-Japan Friendship Hospital, Beijing, China; ^2^Graduate School of Peking Union Medical College, Beijing, China; ^3^Peking University China-Japan Friendship School of Clinical Medicine, Beijing, China

**Keywords:** intraplaque hemorrhage, immune cell infiltration, bioinformatics, GEO, atherosclerosis

## Abstract

**Background:**

Intraplaque hemorrhage (IPH) is an important feature of unstable plaques and an independent risk factor for cardiovascular events. However, the molecular mechanisms contributing to IPH are incompletely characterized. We aimed to identify novel biomarkers and interventional targets for IPH and to characterize the role of immune cells in IPH pathogenesis.

**Methods:**

The microarray dataset GSE163154 which contain IPH and non-IPH plaque samples was obtained from the Gene Expression Omnibus (GEO). R software was adopted for identifying differentially expressed genes (DEGs) and conducting functional investigation. The hub genes were carried by protein-protein interaction (PPI) network and were validated by the GSE120521 dataset. CIBERSORT deconvolution was used to determine differential immune cell infiltration and the relationship of immune cells and hub genes. We confirmed expression of proteins encoded by the hub genes by immunohistochemistry and western blotting in 8 human carotid endarterectomy samples with IPH and 8 samples without IPH (non-IPH).

**Results:**

We detected a total of 438 differentially expressed genes (DEGs), of which 248 were upregulated and 190 were downregulated. DEGs were mainly involved in inflammatory related pathways, including neutrophil activation, neutrophil degranulation, neutrophil-mediated immunity, leukocyte chemotaxis, and lysosomes. The hub genes found through the method of degree in the PPI network showed that *ITGB2* and *ITGAM* might play an important role in IPH. Receiver operating characteristic (ROC) results also showed a good performance of these two genes in the test and validation dataset. We found that the proportions of infiltrating immune cells in IPH and non-IPH samples differed, especially in terms of M0 and M2 macrophages. Immunohistochemistry and western blotting analysis showed that expression levels of *ITGB2* and *ITGAM* increased significantly in carotid atherosclerotic plaques with IPH.

**Conclusion:**

*ITGB2* and *ITGAM* are key hub genes of IPH and may play an important role in the biological process of IPH. Our findings advance our understanding of the underlying mechanisms of IPH pathogenesis and provide valuable information and directions for future research into novel targets for IPH diagnosis and immunotherapy.

## Introduction

Carotid atherosclerotic disease is a key risk factor for ischemic stroke, which remains an important cause of mortality and disability worldwide (1). Improvement of atherosclerotic imaging capabilities revealed important new insights, suggesting that the vulnerability of atherosclerotic plaques depends more on their composition than on their size or degree of lumen narrowing (2). Intraplaque hemorrhage (IPH), lipid-rich necrotic cores, thin fibrous caps, and inflammation are considered important features of high-risk atherosclerotic lesions (3). In particular, there is a well-established relationship between IPH and adverse cardiovascular outcomes. Recent studies confirmed that IPH is an independent risk factor for stroke and coronary heart disease, and that the risk of ipsilateral ischemic events in existing IPH patients is increased 4 to 12 times (4–6).

IPH is thought to originate from new, immature vessels that respond to hypoxia or inflammatory stimuli (7, 8). During plaque advancement, intraplaque angiogenesis provides oxygen and nourishment to maintain plaque growth. However, these neovessels are usually immature, characterized by increased permeability caused by a discontinuous basement membrane, underdeveloped interendothelial connections and poor pericyte coverage (9–11). Leaky neovessels tend to rupture and permit extravasation of blood components such as erythrocytes, inflammatory cells, lipoproteins, and plasma, resulting in IPH (9). IPH rapidly enlarges the volume of the necrotic core of the plaque and promotes deposition of free cholesterol by accumulating cholesterol-rich erythrocyte membranes. These processes trigger an inflammatory response and initiate a vicious cycle that destabilizes atherosclerotic plaques (12, 13). Further, accumulated erythrocytes can attract inflammatory cells to exudate from neovessels and release a large amount of cytokines, growth factors, and matrix metalloproteinases (MMPs), thus creating a highly immune-responsive environment and further triggering the formation of new immature intraplaque microvessels (14, 15).

While histopathological and experimental studies improved our understanding of the pathogenesis of IPH, the molecular mechanisms remain unclear. Toward filling this gap, recent advances in gene chip technology have helped identify new and important genes related to disease mechanisms. However, although bioinformatics studies have described gene expression and immune cell infiltration patterns during atherosclerosis occurrence and progression, there are few studies comparing atherosclerotic plaques in tissues with and without IPH (non-IPH) (16–18).

**Figure 1:**
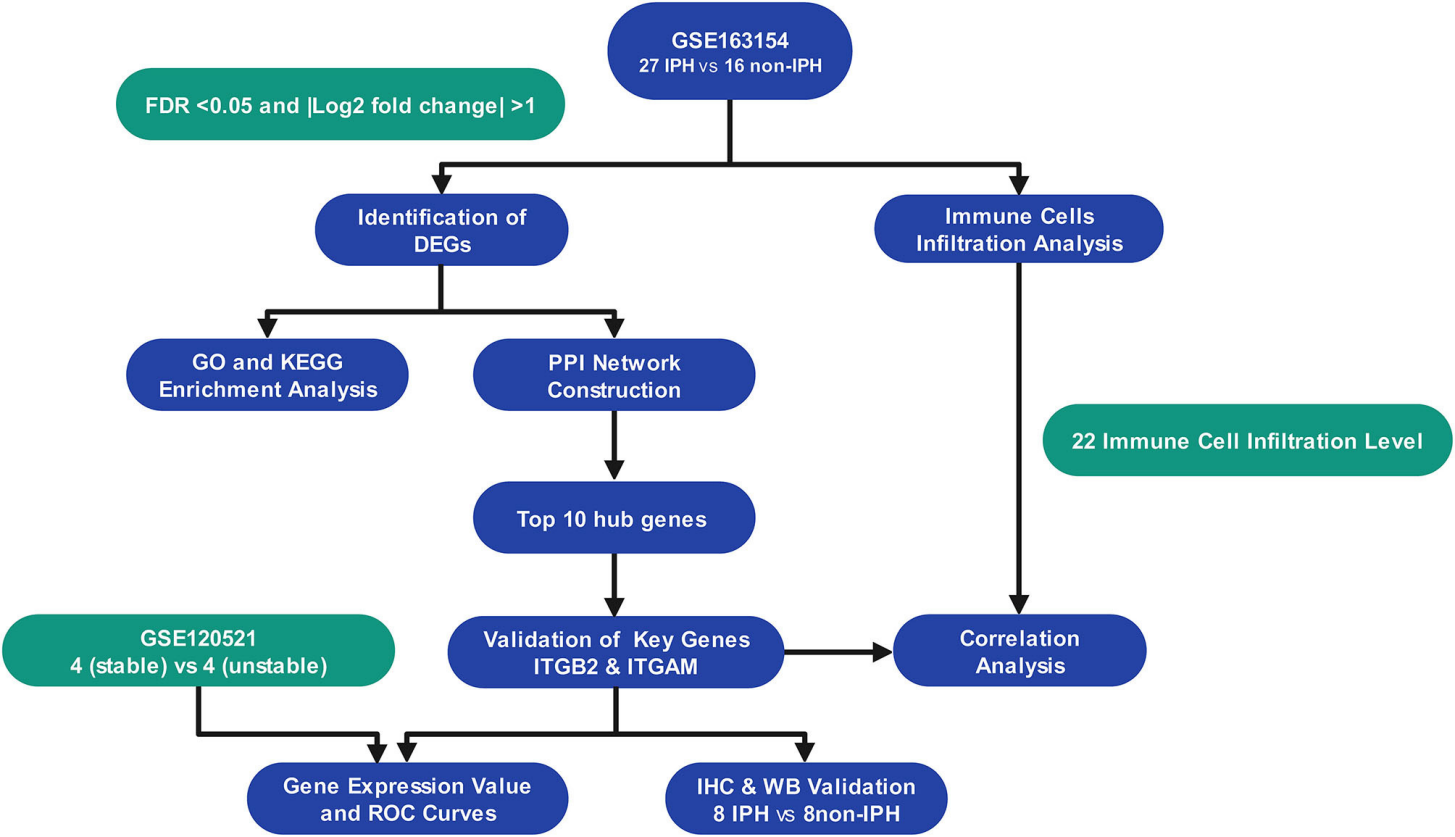
The flowchart of the study.

As the flowchart showed in Figure 1, we downloaded array dataset GSE163154 from the Gene Expression Omnibus (GEO) to study differentially expressed genes (DEGs) and pathways between IPH and non-IPH carotid plaques by bioinformatics methods including DEG screening, functional enrichment analysis, protein–protein interaction (PPI) analysis and identification of hub genes. The GSE120521 dataset was used to validate the hub genes and CIBERSORT was used to further analyze immune cell infiltration in these two types of plaques. Finally, the protein expression associated with the important hub genes was verified in carotid endarterectomy specimens by immunochemical staining and western blotting. Our results contribute to understanding the molecular mechanisms underlying IPH development and highlight the importance of immune cells in the pathogenesis of IPH.

## Materials and Methods

### Microarray Data

mRNA expression profiles of GSE163154, which were assayed on the GPL6104 platform (Illumina humanRef-8 v2.0 expression beadchip), were obtained from the GEO database. The dataset contains 43 carotid atherosclerotic plaques, including 16 non-IPH plaque samples and 27 IPH plaque samples, which were collected from symptomatic patients undergoing carotid endarterectomy (CEA) surgery. Meanwhile the dataset GSE120521 obtained from the GEO was used as an external validation dataset, including 4 stable plaques (macroscopically normal) and 4 unstable plaques (plaque rupture with IPH).

### Test for Correlation and Variation of Samples

Pearson's correlation analysis and principal component analysis (PCA) were performed for the mRNA expression profile in GSE163154 dataset to examine the correlation and variation of the samples. All statistical computing and graphics were performed using R software. Pearson's correlation test was used to evaluate the correlation among all samples, and a correlation heatmap was drawn to visualize correlations between samples using the pheatmap package of R (version 4.1.0, https://www.r-project.org/). PCA was used to visualize the variation and clustering of samples. If the samples within the group can be clustered or have high correlation, the data was considered to have good quality and reliability for bioinformatics analysis.

### Identification of DEGs

The limma package in R was used to normalize and screen DEGs between non-IPH samples and IPH samples. DEGs with an adjusted false discovery rate (FDR) *p* < 0.05 and |log2 fold change (FC)| >1 were considered significant. A heatmap was drawn using the pheatmap package for visualizing DEGs.

### Enrichment Analysis

Gene Ontology (GO) term and Kyoto Encyclopedia of Genes and Genomes (KEGG) pathway enrichment analyses were performed for DEGs using the clusterProfiler package, and the *p*-value cutoff and q-value cutoff were set to 0.05.

### Construction of the PPI Network and Identification of Hub Genes

The online Search Tool for the Retrieval of Interacting Genes (STRING, https://www.string-db.org/) database was used to construct the DEG PPI network, with a PPI score threshold (medium confidence) ≥0.4. The Cytohub plugin in Cytoscape (version 3.8.2, https://cytoscape.org/) was used to identify hub genes using the degree method (Top 10 genes). Moreover, we verified the expression of the crucial genes and evaluated the accuracy of crucial genes using receiver operating characteristic (ROC) curves in internal dataset GSE163154 and external dataset GSE120521.

### CIBERSORT Analysis of Immune Cell Infiltration

The CIBERSORT (https://cibersortx.stanford.edu/) deconvolution algorithm was used to evaluate differential immune cell infiltration between IPH and non-IPH samples. CIBERSORT is an analysis tool that uses gene expression data to estimate the abundances of member cell types in a mixed cell population. The LM22 gene file provided by CIBERSORT was used to define and infer the relative proportions of 22 types of infiltrating immune cells in the IPH and non-IPH plaque gene expression data.

The default signature matrix of 100 permutations was used in this algorithm. To ensure confidence in the results, CIRBERSORT uses Monte Carlo sampling to derive the deconvolution *p*-value for each sample, and only data with *p*-values <0.05 were retained. After data processing and filtering, 14 cases of non-IPH data and 27 cases of IPH data were included in the subsequent analysis. The results obtained by CIBERSORT were visualized using the corplot, vioplot, and ggplot2 packages in R. We then performed correlation analysis between the 22 immune cells and the key genes using Spearman's rank correlation test.

### Sample Collection and Classification

From December 2020 to September 2021, we collected 37 carotid plaques during CEA surgery in China-Japan Friendship Hospital. Through preliminary macroscopic observation, we intentionally selected 8 IPH plaques and 8 non-IPH plaques for analyze and retrospectively collected clinical characteristic information of patients. The collected specimens were cut into 5–8-mm-thick parallel sections, and each alternate section was quickly frozen in liquid nitrogen and stored at −80°C for subsequent protein extraction, while the rest of the sections were fixed in 4% polyoxymethylene for 24 h, and then embedded in paraffin. Histological examination was performed on 5-mm-thick serial sections. Sections from different segments of each sample were taken for hematoxylin-eosin (H&E) and Perls staining (Solarbio, G1424) to reconfirm the presence of IPH. This study was approved by the Medical Ethics Committee of the China-Japan Friendship Hospital of Beijing, China (2019-25-1), and we received informed consent from all patients.

### Immunohistochemistry

Sections were deparaffinated, blocked, and incubated with the primary anti-*ITGB2* antibody (Proteintech, 10554-1-AP) or anti-*ITGAM* antibody (Proteintech, 21851-1-AP) at 4°C overnight. Image-Pro Plus 6.0 software (IPP 6.0, Media Cybernetics, United States) was utilized to measure the total tissue area and integrated optic density (IOD) of the target gene, which was stained yellow-brown. The intensity of gene expression was presented as IOD per unit area.

### Protein Extraction and Western Blotting Analysis

Plaque samples were washed twice with cold phosphate-buffered saline and lysed with RIPA buffer (Beyotime Technology; Cat: P0013C) containing proteinase inhibitors. Total protein concentrations were measured using a BCA Protein Assay Kit (Invitrogen; Cat: 23227). Equal amounts of protein were separated by SDS-PAGE and transferred to a PVDF membrane. Blot membranes were blocked with 5% non-fat milk, and incubated with primary antibodies (*ITGAM*, 1:2000, Proteintech; *ITGB2*, 1:1000, Proteintech) followed by suitable peroxidase-conjugated secondary antibody. Immunoreactive bands were detected with Pierce ECL Western Blotting Substrate (Thermo Scientific; Cat: 32209). β-actin was used as an internal control and blots were quantified by Image J.

### Statistical Analysis

R version 4.1.0 was used to perform bioinformatics analyses and a *p*-value or adjusted *p*-value < 0.05 was considered statistically significant. SPSS version 26 and GraphPad Prism 6.0 software were used to analyze clinical and experimental data. Unpaired Student's *t*-test was used to compare the two sets of data. *P* < 0.05 was considered statistically significant.

## Results

### Dataset Validation

**Figure 2:**
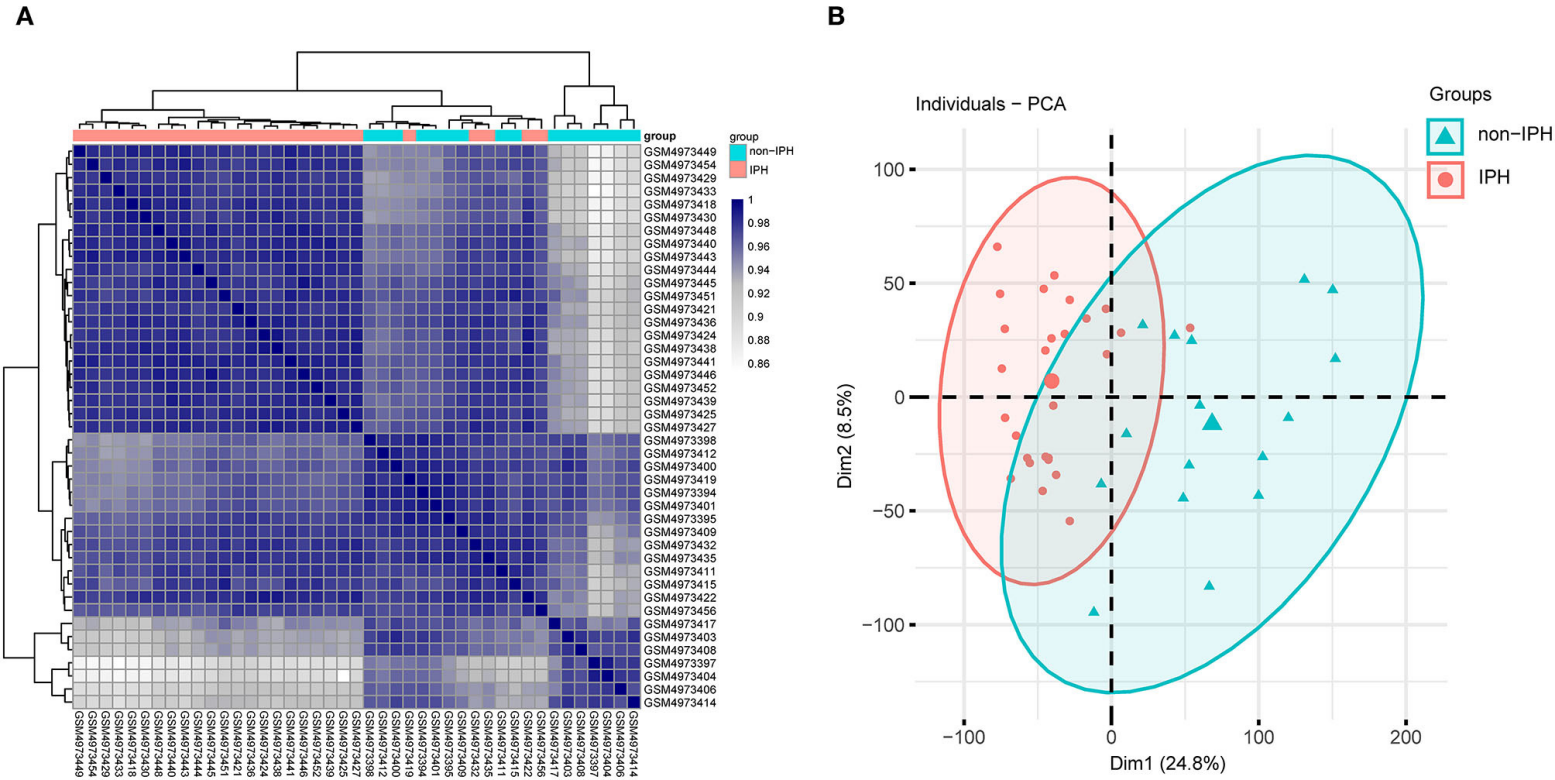
Validation of the dataset GSE163154 by Pearson's correlation analysis and PCA. **(A)** Pearson's correlation analysis of samples from the GSE163154 dataset. The correlation coefficient is reflected by the colors in the heatmap. **(B)** PCA of samples from the GSE163154 dataset. PC1 and PC2 are represented on the x-axis and y-axis, respectively. PCA, principal component analysis; PC1, principal component 1; PC2, principal component 2.

Pearson's correlation test and PCA were used to validate the dataset. The correlation heatmap of the GSE163154 dataset showed that there were strong correlations among samples within the IPH group and also between samples within the non-IPH group (Figure 2A). PCA of GSE163154 showed that the 43 samples in the two groups could be distinguished, as the distances between the samples in the IPH group were close in the dimensions of PC1 and PC2 and the distance between samples in the non-IPH group were also close (Figure 2B).

### Identification of DEGs

**Figure 3:**
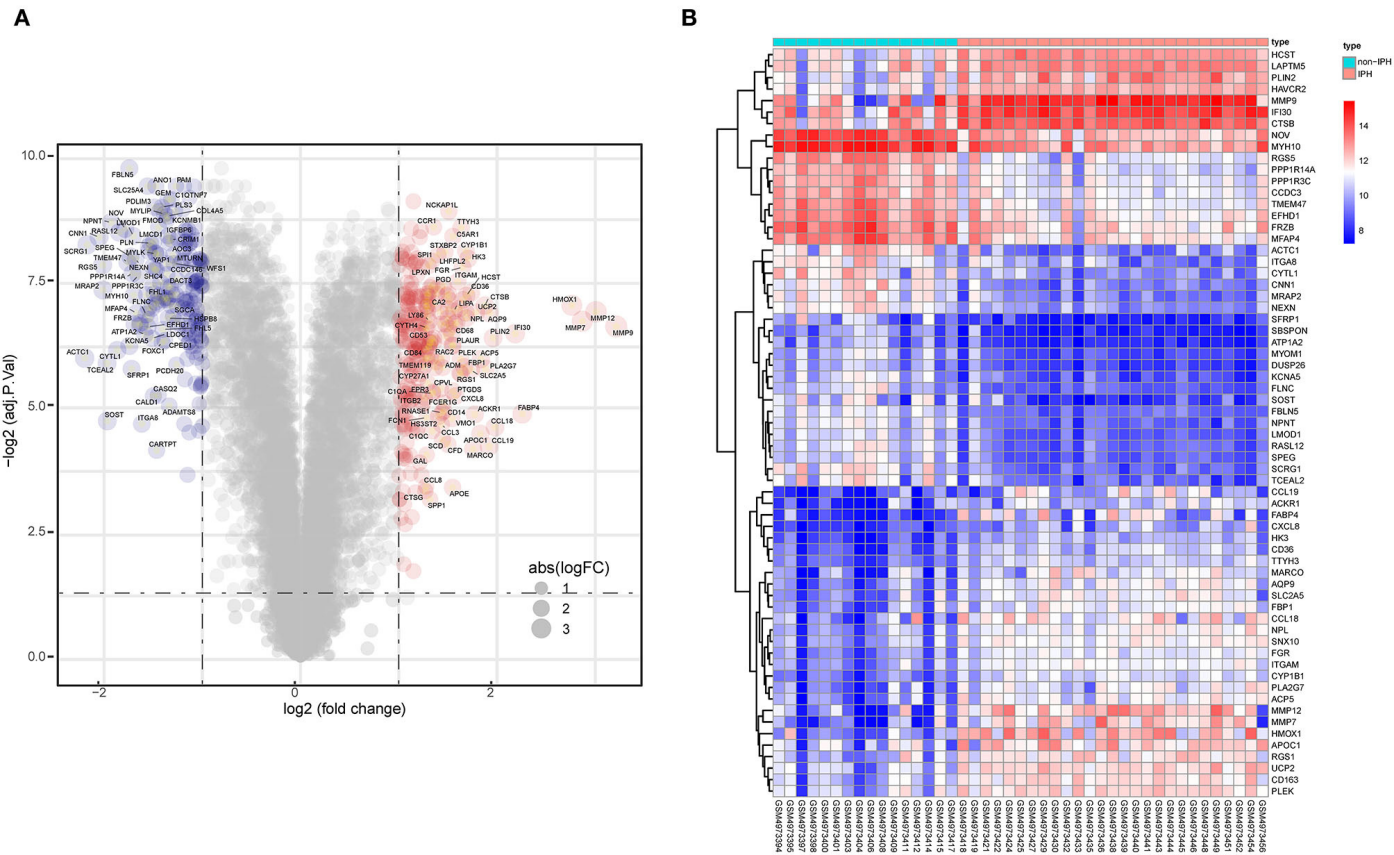
Identification of DEGs between non-IPH samples and IPH samples. **(A)** Volcano plot showing the DEGs between non-IPH and IPH groups after analysis of the GSE163154 dataset with R software. The x-axis represents the fold-change (log-scaled) and the y-axis represents the *p*-value (log-scaled). Red symbols represent upregulated genes, blue symbols represent downregulated genes, and gene name symbols represent the DEGs with the greatest magnitude of fold-change. **(B)** A heatmap showing the DEGs between the two groups. Upregulated genes are labeled in red and downregulated genes are shown in blue.

A total of 438 DEGs were screened, with an adjusted *p*-value of <0.05 and | log2 (fold-change) | >1 as thresholds. A total of 248 upregulated and 190 downregulated DEGs were identified in IPH samples when compared to non-IPH samples, as shown by volcano plot (Figure 3A) and heatmap (Figure 3B), while a detailed summary was listed in Supplementary Table S1.

### Functional and Pathway Enrichment Analysis

**Figure 4:**
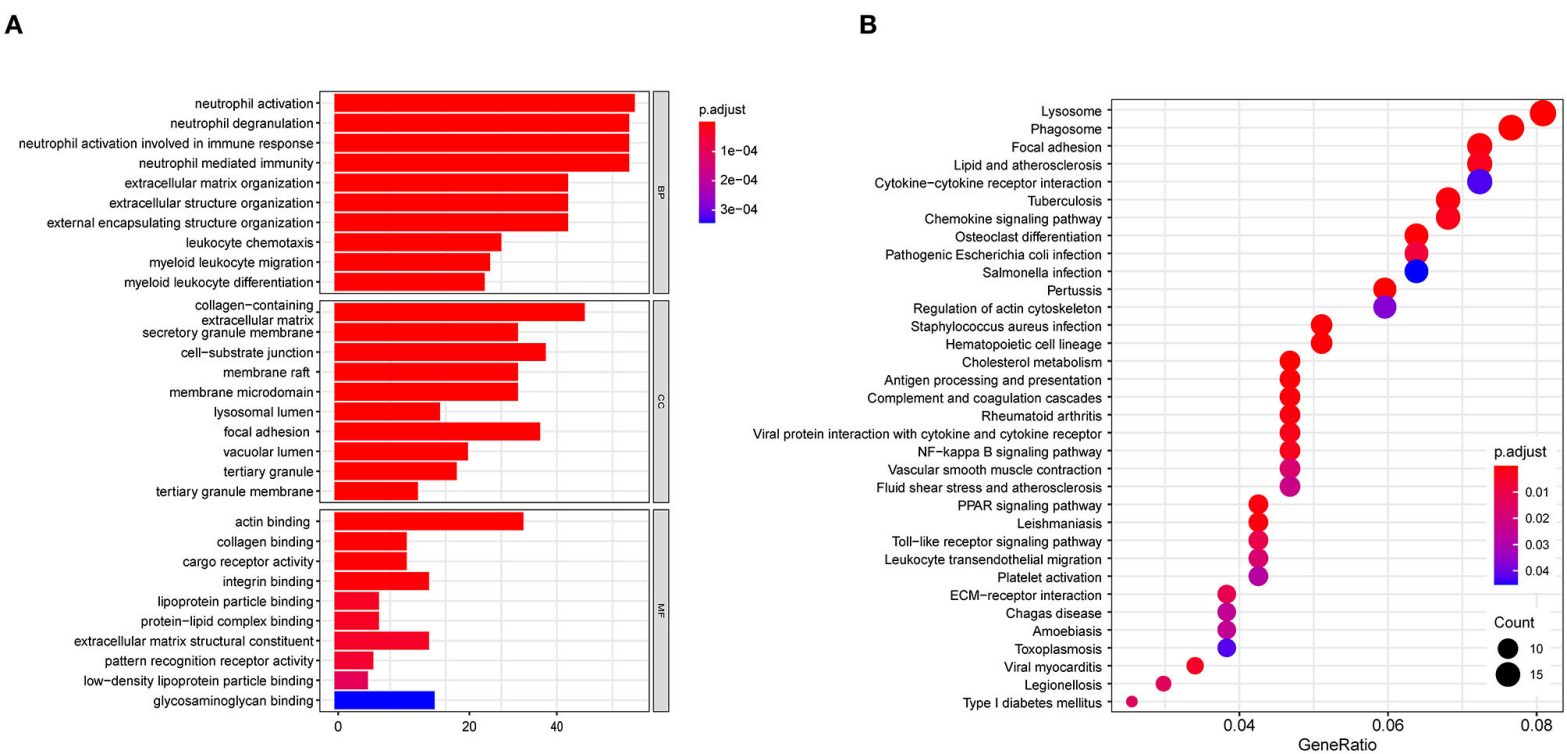
Biofunctional enrichment analysis of DEGs. **(A)** GO functional enrichment analyses of DEGs and the top 10 BP, CC, and MF terms (BP, Biological Process; CC, Cellular Component; MF, Molecular Function). **(B)** KEGG pathway enrichment analyses of DEGs.

GO analysis classified DEGs into three categories: biological process (BP), molecular function (MF), and cellular component (CC). DEGs linked with BP were significantly enriched in neutrophil activation, neutrophil degranulation, and neutrophil activation involved in immune response. DEGs linked with CC were significantly enriched in collagen-containing extracellular matrix, secretory granule membrane, and cell-substrate junction. DEGs linked with MF were significantly enriched in actin binding, collagen binding, and cargo receptor activity (Figure 4A). KEGG pathway enrichment analysis revealed that DEGs were mainly enriched in lysosome, pertussis, cholesterol metabolism, and phagosome pathways (Figure 4B). The detatiled results were listed in Supplementary Table S2.

### Construction of the PPI Network and Screening of Hub Genes

**Figure 5:**
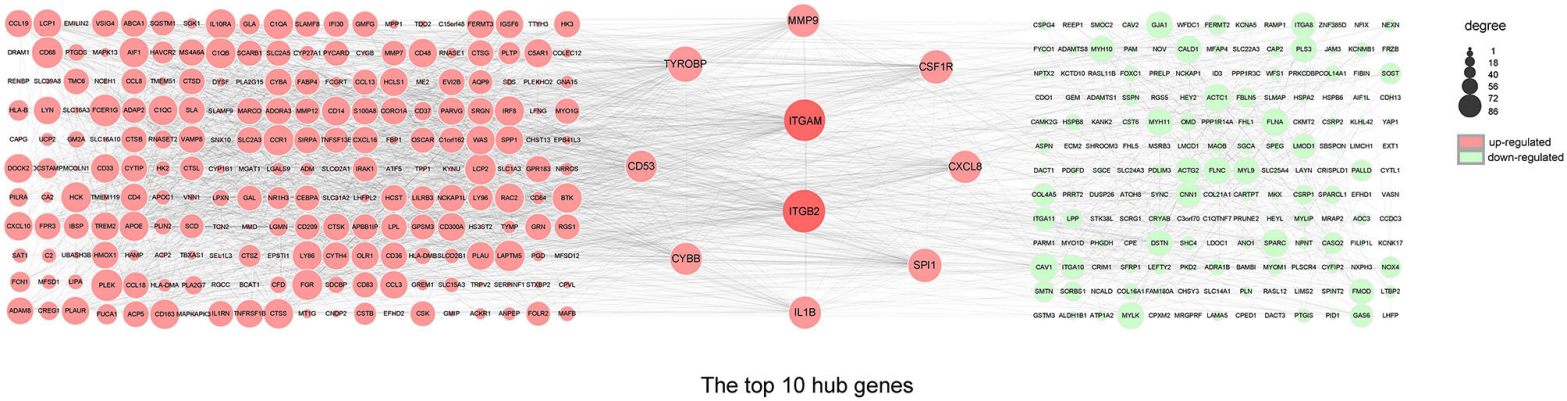
PPI network and hub genes. The PPI network analyzed by the STRING database. The top 10 hub genes screened by the degree method using CytoHubba plugin (Cytoscape). The size of the circle from small (lower) to large (higher) represents the ranking. Red and green represent upregulated and downregulated genes, respectively. DEGs, differentially expressed genes; *ITGB2*, integrin subunit beta 2; *ITGAM*, integrin subunit alpha M; *TYROBP*, transmembrane immune signaling adaptor TYROBP; *SPI1*, Spi-1 proto-oncogene; *CSF1R*, colony stimulating factor 1 receptor; *MMP9*, matrix metallopeptidase 9; *CXCL8*, C-X-C motif chemokine ligand 8; *IL1B*, interleukin 1 beta; *CYBB*, cytochrome b-245 beta chain; *CD53*, CD53 molecule.

The top ten genes *ITGB2, ITGAM*, TYROBP, SPI1, CSF1R, MMP9, CXCL8, IL1B, CYBB, and CD53 obtained by PPI analysis and Cytoscape were regarded as hub genes, of which *ITGB2* and *ITGAM* were in the most critical positions and became the focus of subsequent analyses (Figure 5).

### Different Immune Cell Infiltrative Patterns Between IPH and Non-IPH Samples

**Figure 6:**
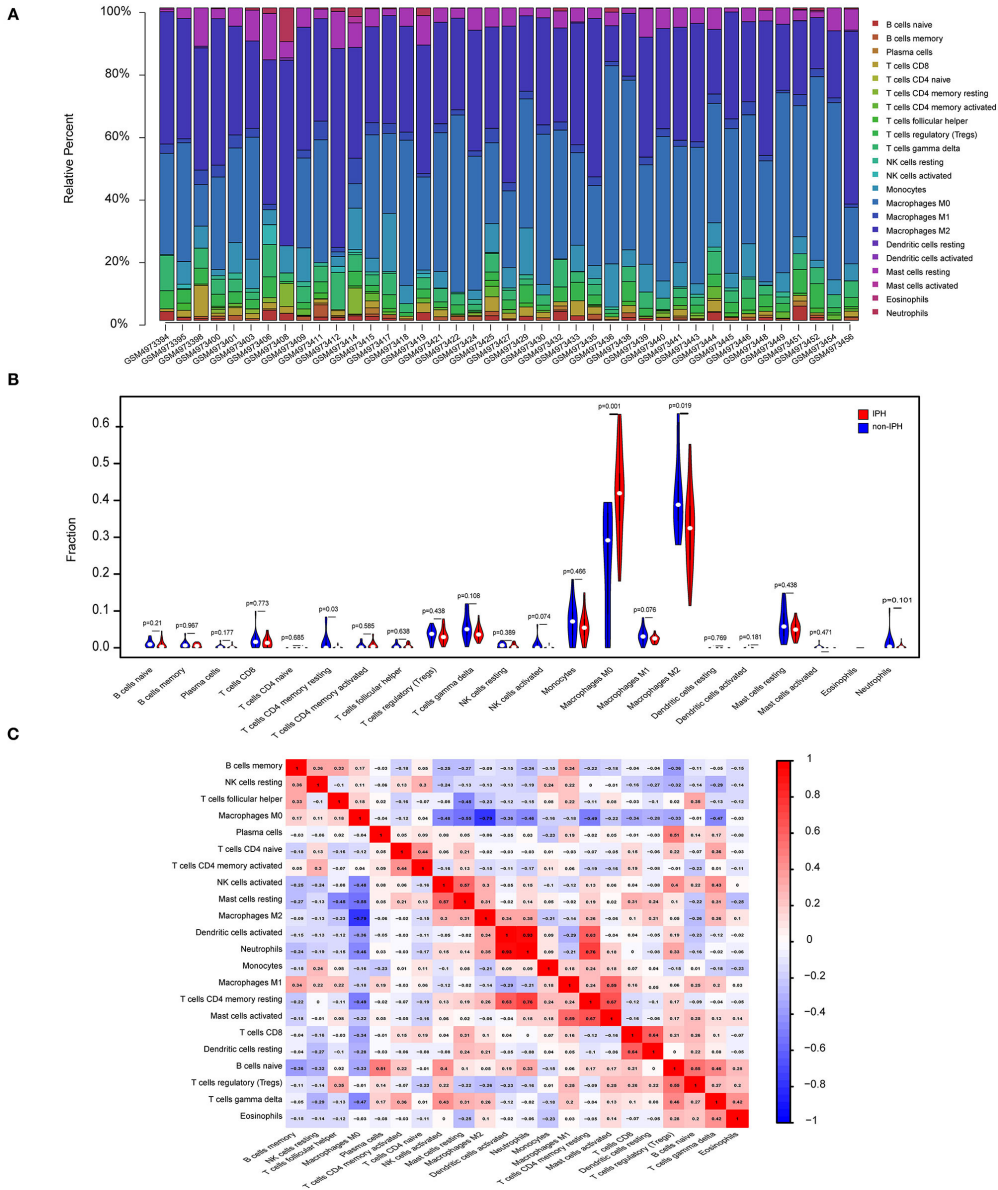
Immune cell infiltration patterns in IPH samples and non-IPH samples. **(A)** Histogram of the proportions of 22 immune cell subpopulations in each IPH and non-IPH sample. x-axis: GEO samples; y-axis: percentage of each immune cell type. **(B)** Violin plot showing the differentially infiltrated immune cells between the two groups. Blue represents the non-IPH plaque group and red represents the IPH plaque group. **(C)** Correlation heatmap of all immune cells. Numbers in the small square represent Pearson's correlation coefficient between the two immune cells on the horizontal and vertical coordinates; red squares indicate positive correlation, and blue squares indicate negative correlation.

GO and KEGG analysis identified multiple pathways related to the immune process. Therefore, we used CIBERSORT software to reveal the pattern of immune cell infiltration in carotid atherosclerotic plaques with hemorrhage. After data processing and screening, 14 cases of non-IPH data and 27 cases of IPH data were included in the subsequent analysis, and a heatmap was used to show the proportion of 22 immune cells in these two groups of samples (Figure 6A). M2 macrophages, M0 macrophages, resting mast cells, gamma delta T cells, and monocytes represented the top five highest infiltrating fractions in both groups of plaques. Compared with the non-IPH group, the proportion of M0 macrophages was higher in the IPH group, while the proportion of M2 macrophages was lower (Figure 6B). Furthermore, we performed a correlation analysis of infiltrated immune cells in the plaques, with scores representing the degree of correlation (Figure 6C). The correlation heatmap indicated that activated dendritic cells and neutrophils showed the most synergistic effect, while M0 macrophages and M2 macrophages showed the most competitive effect.

### Analysis Between Crucial Genes and Immune Cells

**Figure 7:**
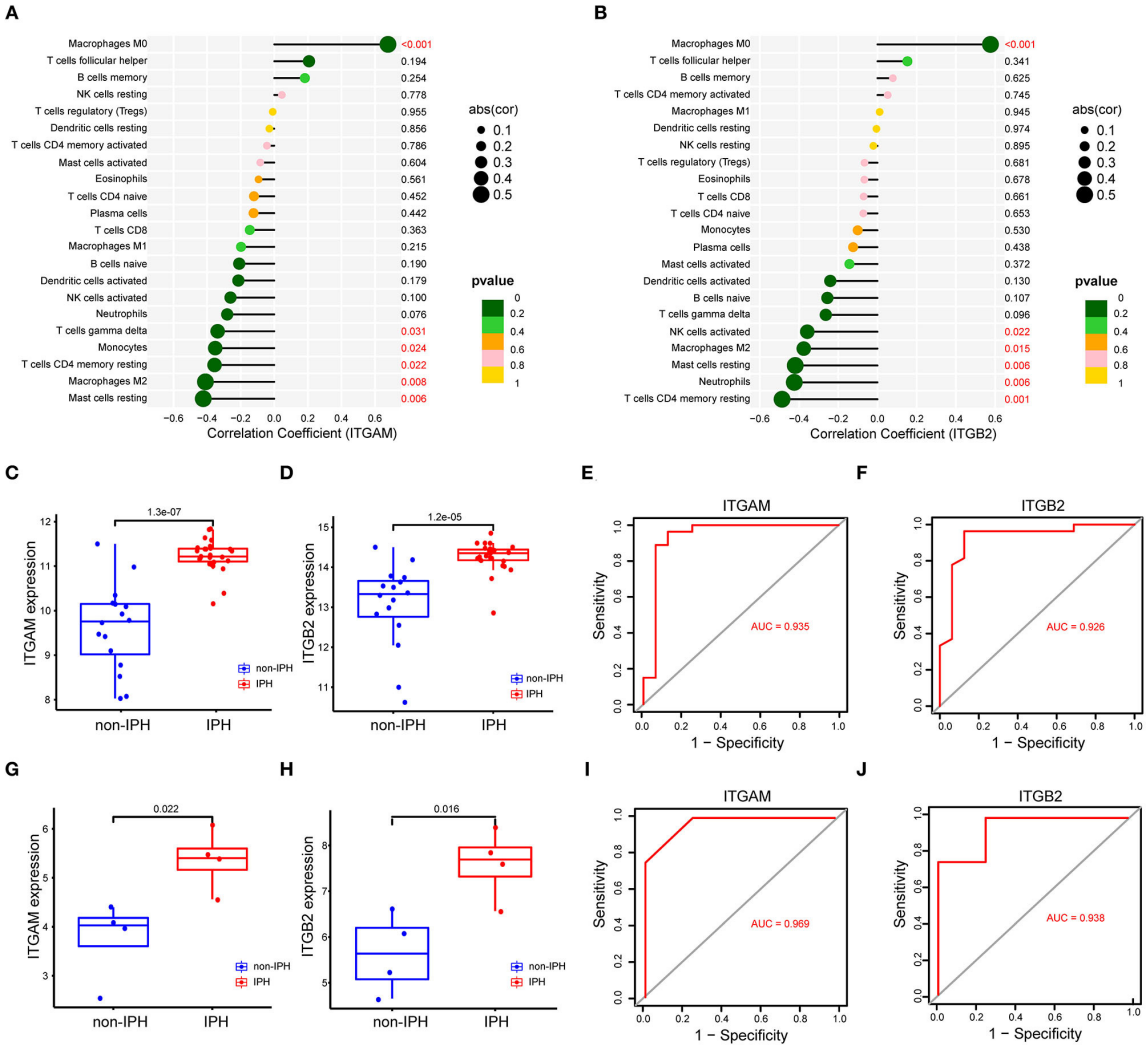
**(A,B)** Correlation between the hub genes (*ITGAM, ITGB2*) and infiltrating immune cells. The size of the dots represents the strength of the correlation between genes and immune cells, and the color of the dots represents the *p*-value. *p* < 0.05 was considered statistically significant. **(C,D)** The expression level of two genes between IPH and non-IPH samples in dataset GSE163154. **(E,F)** ROC curves for evaluating the accuracy of logistic regression analysis of dataset GSE163154. **(G,H)** The expression level of two genes between IPH and non-IPH samples in validation dataset GSE120521. **(I,J)** ROC curves for evaluating the accuracy of logistic regression analysis of validation dataset GSE120521. ROC, receiver operating characteristic.

As indicated from the correlation analysis, *ITGAM* displayed a significant positive correlation with M0 macrophages (r = 0.678, *p* < 0.001), and a significant negative correlation with resting mast cells (r = −0.423, *p* = 0.006), M2 macrophages (r = −0.410, *p* = 0.008), and resting mast cells (r = −0.423, *p* = 0.006) (Figure 7A). *ITGB2* displayed a significant positive correlation with M0 macrophages (r = 0.576, *p* < 0.001), and a significant negative correlation with resting mast cells (r = −0.419, *p* = 0.006), neutrophils (r = −0.424, *p* = 0.006), as well as CD4 memory resting T cells (r = −0.487, *p* = 0.001) (Figure 7B). A detailed summary was listed in Supplementary Table S3.

### Internal and External Validation of Key Genes

We validated expression of the two key genes and performed ROC analysis on internal dataset GSE163154 and external dataset GSE120521. Results revealed that *ITGAM* and *ITGB2* were significantly upregulated in IPH or advanced atheroma plaques (Figures 7C,D,G,H). The ROC curves revealed the probability of *ITGAM* and *ITGB2* as valuable biological markers with AUCs of 0.935 and 0.926 (Figures 7E,F); the ROC analysis of external data sets also showed good diagnostic effects of *ITGAM* and *ITGB2*, with AUCs of 0.820 and 0.825, respectively (Figures 7I,J). Differences in expression levels of the remaining 8 hub genes (TYROBP, SPI1, CSF1R, MMP9, CXCL8, IL1B, CYBB, and CD53) and their correlation with immune cells were detected in dataset GSE163154 (Supplementary Figure S1).

### Demographic Data of the Patients and the Expression of *ITGB2* and *ITGAM*

**Figure 8:**
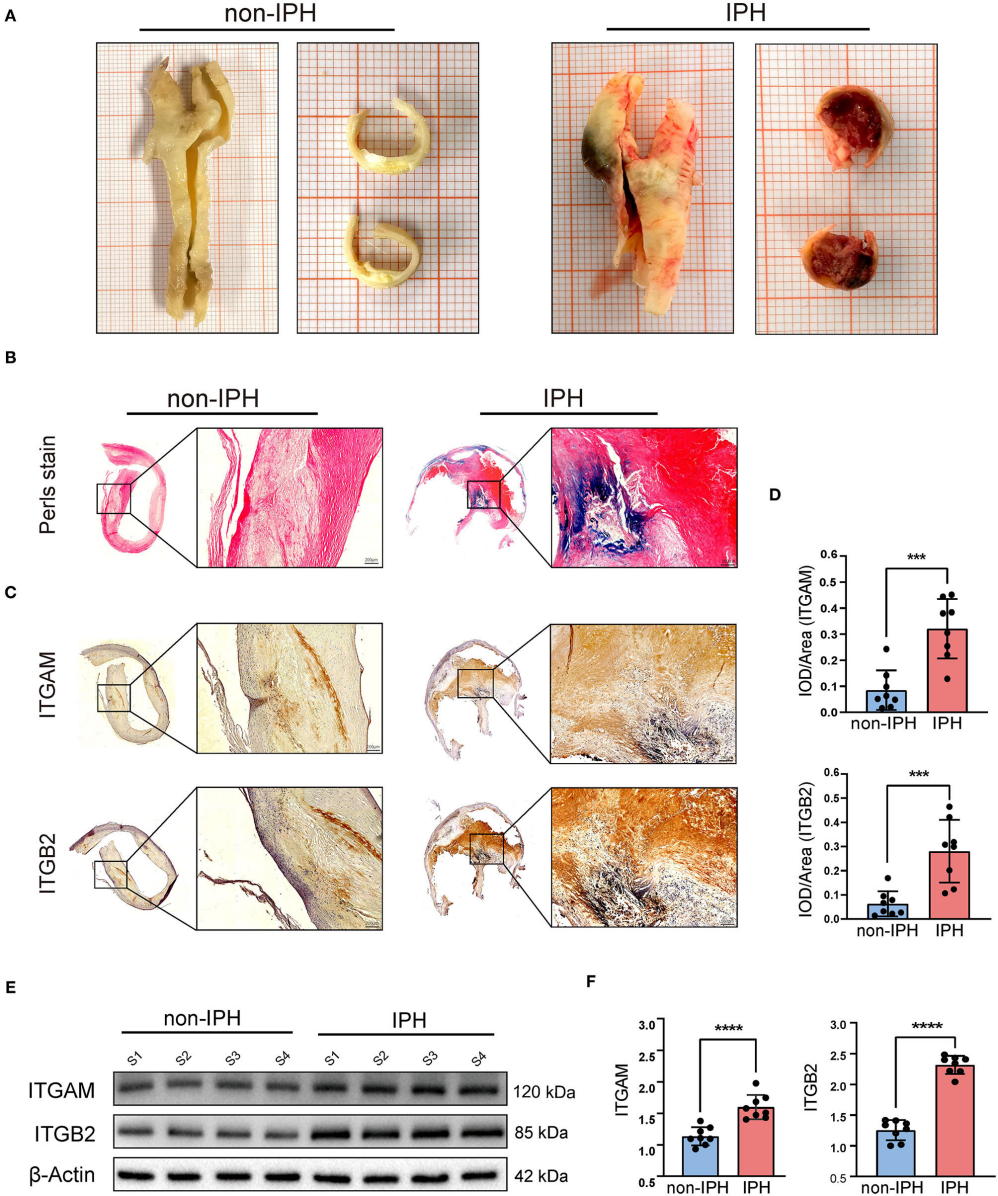
Validation of the hub genes (*ITGAM* and *ITGB2*). **(A)** Fresh human carotid atherosclerotic plaque specimens were collected from carotid endarterectomy. Plaque features can be preliminarily observed in gross specimens and cross sections. From left to right, atherosclerotic plaques without IPH and with IPH are shown. **(B)** Perls staining was used to further identify the plaques. The IPH plaque section showed obvious blue staining and infiltration of erythrocytes by light microscopy. **(C)** Typical micrographs of *ITGAM* and *ITGB2* immunohistochemical staining, macroscopic, and microscopic views of the cross sections from left to right. **(D)** Histograms show the quantitative results of immunohistochemical staining. *ITGAM* and *ITGB2* expression were significantly increased in the IPH group. **(E)** Western blotting was used to determine the protein expression levels. Quantitative results **(F)** show that the expression of *ITGB2* and *ITGAM* in the IPH group was significantly higher than that in the non-IPH group. ***p < 0.001, ****p < 0.0001.

The plaques were divided into IPH plaques and non-IPH plaques by macroscopic examination (Figure 8A), H&E and Perls staining of tissue sections. Perls staining showed hemosiderin in hemorrhagic plaques in blue color and revealed the accumulation of erythrocytes in the hemorrhagic area within a plaque (Figure 8B). After screening and pathologic confirmation, 8 IPH plaques and 8 non-IPH plaques were intentionally selected for subsequent analysis. Patients' clinical characteristics including age, gender, body mass index (BMI), hypertension, diabetes mellitus, coronary heart disease (CHD), serum total cholesterol (TC), and smoking were retrospectively collected and compared between groups. None of the clinical characteristics differed significantly between the two groups (Table 1).

**Table 1:** Patient demographic data.

	**Total**	**Non-IPH**	**IPH**	***p*-Value**
Patients	16	8	8	–
Male (*n* [%])	11 (68.7%)	5 (62.5%)	6 (75%)	1
Age (years)	65.31 ± 8.42	65.00 ± 8.452	65.63 ± 8.975	0.888
BMI (kg/m^2^)	27.31 ± 1.968	27.977 ± 1.966	26.655 ± 1.854	0.188
Hypertension Yes	13 (81.2%)	7 (87.5%)	6 (75%)	1
Diabetes mellitus Yes	7 (43.7%)	2 (25%)	5 (62.5%)	0.315
CHD Yes	3 (18.7%)	0 (0%)	3 (37.5%)	0.2
TC (mmol/L)	3.48 ± 0.43	3.332 ± 0.240	3.63 ± 0.537	0.181
Smoker Yes	10 (62.5%)	4 (50%)	6 (75%)	0.608

*Values are shown as mean ± SD or n (%). IPH, intraplaque hemorrhage; BMI, body mass index; CHD, coronary heart disease; TC, serum total cholesterol (TC)*.

Immunohistochemistry was used to assess *ITGB2* and *ITGAM* expression and tissue distribution. *ITGB2* and *ITGAM* expression was significantly higher in IPH samples, especially in the hemorrhagic area (Figure 8C). Quantitative analysis by IPP software showed that the mean DOI of the tissue area (DOI/Area) of IPH samples was significantly higher than that of non-IPH samples (Figure 8D). Western blotting was used to evaluate the protein expression levels of *ITGB2* and *ITGAM* in 8 samples from each group, and band densities were quantified using Image J software. The expression levels of the two molecular proteins in the IPH group were significantly higher than those in the non-IPH group (Figures 8E,F).

## Discussion

Increasing evidence indicates that IPH is associated with high risk of atherosclerotic plaques. In fact, IPH is not only a marker of unstable plaques, but also a trigger of plaque instability (13). IPH leads to a series of subsequent pathological processes, such as accumulation of cholesterol-rich erythrocyte membranes (19), expansion of necrotic cores, promotion of oxidant and proteolytic activity, infiltration of leukocytes, and a highly inflammatory plaque environment (8, 20, 21). Therefore, preventing the occurrence and progression of IPH is of great significance for increasing plaque stability and preventing stroke. However, IPH is a complex multifactorial disease with unclear pathologic mechanisms, and valuable biomarkers are needed to predict and prevent IPH-related stroke.

The key hub genes identified in this study, *ITGB2* (also known as *CD18*) and *ITGAM* (also known as *CD11b*), belong to the integrin family, which is an important transmembrane protein family that mediates cell-cell adhesion and cell-extracellular matrix (ECM) adhesion (22). Previous studies have demonstrated that *ITGAM* (integrin αM) and *ITGB2* (integrin β2) can promote leukocyte transendothelial migration and disrupt endothelial barrier function through animal and cultured cells experiments (23, 24), and some studies have reported that *ITGB2* and *ITGAM* may be involved in the progression of atherosclerosis through bioinformatics analysis (25, 26). However, few studies have directly reported the relationship between these two genes and IPH. Different from the *in vivo* and *in vitro* experimental methods in previous studies, and also different from the genomic analysis of unclassified atherosclerotic plaques, this study directly conducted bioinformatics analysis on high-throughput gene chip data of human IPH plaques. Through differential gene screening, PPI analysis and ROC verification, *ITGAM* and *ITGB2* were identified as key hub genes of IPH, and further histological examination proved for the first time that *ITGB2* and *ITGAM* were highly expressed in IPH plaques. In addition, we revealed for the first time the correlations between *ITGB2/ITGAM* and various types of immune cells in atherosclerosis plaques by immune cell infiltration analysis. These results provided a new comprehensive perspective for understanding the pathogenesis of IPH and provided valuable clues for finding potential therapeutic targets for IPH and IPH-related stroke.

*ITGB2* (also known as *CD18*) and *ITGAM* (also known as *CD11b*) encode the integrin β2 subunit and αM subunit, respectively. Integrins are heterodimers formed by specific non-covalent binding of α and β subunits, and have traditionally been considered important regulators of cell survival, proliferation, adhesion, and migration (22, 27). *ITGB2* encodes the integrin β2 subunit, which is non-covalently coupled to different α subunits to form the β2 integrin family (including αLβ2, αMβ2, αXβ2, and αDβ2) (28). β2 integrin is a major receptor family on many leukocyte subsets and plays an important adhesion function in the process of leukocyte recruitment, antigen presentation, pathogen clearance and thrombosis (29). In addition, recent studies showed that β2 integrin controls various cellular metabolic signals and pathways. Zhang et al. reported that *ITGB2* enhances the glycolysis activity of cancer-associated fibroblasts through the PI3K/AKT/mTOR pathway, thus playing a key role in promoting cancer cell proliferation (30). Furthermore, Liu et al. found that *ITGB2* expression of cancer cells can be induced by YAP to promote cancer cell invasion of cancer cells in a manner similar to that of leukocytes (31).

The integrin αM (CD11b) subunit encoded by *ITGAM* is coupled with the β2 subunit (CD18) to form αMβ2 integrin, also known as Mac-1 (CD11b/CD18). It is expressed mainly on cells of the myeloid lineage, such as monocytes and neutrophils, and certain lymphocyte subsets and is therefore often regarded as a marker of circulating monocytes (32). Mac-1 is involved in phagocytosis, adhesion, and trans-endothelial cell migration, as well as other functions such as regulating apoptosis and degranulation (23, 29). As a major member of the β2 integrin family, CD11b contains an inserted domain that facilitates binding of many ligands, including the adhesion ligands intercellular adhesion molecule-1 and−2 (ICAM-1 and−2), the blood coagulation protein fibrinogen, complement protein iC3b, and the recently discovered MMP9 (33). During leukocyte migration across endothelial cells, Mac-1 promotes adhesion of leukocytes to the ligand ICAM-1 expressed on endothelial cells, and mediates leukocyte crawling on the vascular wall (34, 35). In addition, studies reported that when integrin on immune cells binds to ligands on endothelial cells, it will activate downstream signaling pathways and destroys the intercellular link molecule VE-cadherin (14, 36, 37). This may be a key step in the occurrence of IPH and immune cell infiltration in atherosclerotic lesions.

The results of this study are consistent with previous studies. Meng et al. reported that *ITGB2* and *ITGAM* are involved in the progression of carotid atherosclerotic plaques (25). In addition, high expression of *ITGAM* is associated with unstable atherosclerotic plaques (26), and *ITGAM* knockout reduces macrophage infiltration, MMP9 expression, and elastin and collagen degradation in mouse abdominal aortic aneurysm models (38). This suggests that *ITGAM* and *ITGB2* may play an important role in the occurrence of IPH, immune cell infiltration, and the progression of atherosclerosis.

To further investigate the effect of immune cells in atherosclerotic hemorrhagic plaques, we performed a comprehensive analysis of immune cell infiltration. In this analysis, M2 macrophages, M0 macrophages, resting mast cells, gamma delta T cells, and monocytes represented the top five highest infiltrating fractions in carotid atherosclerotic plaques, which is consistent with previous studies using CIBERSORT analysis and single cell sequencing (16, 39).

Immune cells, especially macrophages, play a critical role in atherogenesis. A recent single-cell sequencing study found that CD4+ T cells, CD8+ T cells, and macrophages dominate the human carotid atherosclerotic plaque immune landscape, while mass-cytometry analysis also revealed two macrophage clusters corresponding to classically activated M1 and alternately activated M2 phenotypes. Nevertheless, plaque macrophages had higher resolution at the single-cell level of transcription, suggesting that these cells have different functional heterogeneity in plaques (39). In a mouse model, aortic atherosclerotic lesions are mainly composed of macrophages, monocytes, and T cells, while the adventitial tissue is dominated by B cells (40). However, few studies have revealed the immune cell landscape of hemorrhagic plaques, so we used CIBERSORT to reveal the differences in infiltrating immune cells between IPH and non-IPH samples. This novel analysis showed a higher proportion of M0 macrophages and a lower proportion of M2 macrophages in IPH samples compared with non-IPH samples. IPH is regarded as a potentially important inflammatory stimulus that promotes macrophage influx into atherosclerotic lesions (41, 42), whereas the reduction of M2 macrophage expression (considered an anti-inflammatory phenotype) in IPH plaques is reasonable (38).

Previous evidence showed that erythrocyte lysis in the IPH region releases free hemoglobin, which can be absorbed by macrophages through CD163 receptors (43). On the other hand, macrophages secrete MMPs and angiogenic factors, including TGF- β, VEGF, and EGF, which undoubtedly further promote the occurrence of new angiogenesis and IPH within plaques (44, 45). This evidence and our results suggest that macrophages and their specific differentiation phenotypes play an important role in intraplaque hemorrhage. However, the specific mechanisms involving macrophages in the process of IPH and the precise signals that trigger macrophage differentiation remain unclear and require further study. Interestingly, the results of this study showed that *ITGB2* and *ITGAM* have positive correlation with M0 macrophages and negative correlation with M2 macrophages. In addition, M0 macrophages were significantly negatively correlated with M2 macrophages, and activated dendritic cells were significantly positively correlated with neutrocytes. These results provide a new direction for future research on the role of macrophages in IPH.

Immunohistochemistry and western blotting showed that the protein expression levels associated with hub genes *ITGB2* and *ITGAM* were significantly higher in the IPH group than in the non-IPH group. Since *ITGAM (CD11b)* is also a marker of macrophages, we concluded that there were more infiltrated macrophages in the IPH group. Notably, immunohistochemistry showed that *ITGB2* and *ITGAM* expression was mainly concentrated in the IPH region or around neovessels (Figure 8C), which may be caused by IPH-induced inflammatory stimulation or neovessel leakage.

These results provide valuable clues for further study on the pathophysiological mechanism of IPH.

This study has several limitations. Firstly, because information on specific clinical characteristics of samples in public datasets could not be collected, we could not rule out the potential impact of heterogeneity in patient populations and clinical characteristics on the results of this study. Secondly, the sample size used for analysis and validation was small, which may affect the accuracy of the analysis results. Future studies need to expand the sample size of IPH plaques prospectively and explore the specific mechanisms of *ITGB2* and *ITGAM* in the development of IPH needs to be further studied through *in vivo* and *in vitro* experiments.

## Conclusion

This study used *in silico* analysis to identify key genes and pathways closely related to the occurrence of IPH. In addition, we described the immune landscape in detail, revealing the underlying immune infiltration patterns of carotid atherosclerotic plaques in the absence or presence of IPH. We validated the key genes *ITGB2* and *ITGAM* experimentally, confirming that the proteins encoded by these genes are highly expressed in IPH plaques. Our findings advance our understanding of the underlying mechanisms of IPH pathogenesis and provide valuable information and directions for future research into novel targets for IPH immunotherapy and diagnosis.

## Data Availability Statement

The datasets presented in this study can be found in online repositories. The names of the repository/repositories and accession number(s) can be found in the article/Supplementary Material.

## Ethics Statement

This study was approved by the Medical Ethics Committee of the China-Japan Friendship Hospital of Beijing, China (2019-25-1). The patients/participants provided their written informed consent to participate in this study.

## Author Contributions

JW and PL designed, guided, and funded the study. XL conducted most of the experimental work. FW, MS, and CS performed the data analysis. XL and BM drafted the manuscript. YY, XF, ZY, JW, and PL critically revised the manuscript. All authors contributed to the article and approved the submitted version.

## Funding

This work was supported by grants from the National Natural Science Foundation of China (nos. 81670275, 81670443, and 82170066) and the International S&T cooperation program (2013DFA31900).

## Conflict of Interest

The authors declare that the research was conducted in the absence of any commercial or financial relationships that could be construed as a potential conflict of interest.

## Publisher's Note

All claims expressed in this article are solely those of the authors and do not necessarily represent those of their affiliated organizations, or those of the publisher, the editors and the reviewers. Any product that may be evaluated in this article, or claim that may be made by its manufacturer, is not guaranteed or endorsed by the publisher.
