# Supplementary material for: Differential Gene Expression and Immune Cell Infiltration in Carotid Intraplaque Hemorrhage Identified Using Integrated Bioinformatics Analysis
Source: Front Cardiovasc Med. 2022 May 17;9:818585. doi: 10.3389/fcvm.2022.818585 (PMC9152291; doi:10.3389/fcvm.2022.818585)
Supplement: Supplementary file 1 [file Table_1.docx]

**Supplementary Table 1: The value of differential gene expression analysis results**

| symbol | logFC | AveExpr | t | P.Value | adj.P.Val |
| --- | --- | --- | --- | --- | --- |
| MMP9 | 3.193382 | 13.42508 | 6.9386 | 1.75E-08 | 2.80E-07 |
| MMP12 | 3.006627 | 11.15154 | 7.284894 | 5.60E-09 | 1.22E-07 |
| MMP7 | 2.86803 | 10.65803 | 7.096243 | 1.04E-08 | 1.90E-07 |
| HMOX1 | 2.754487 | 11.24583 | 7.425023 | 3.54E-09 | 8.89E-08 |
| FABP4 | 2.254321 | 9.655563 | 5.528588 | 1.87E-06 | 1.29E-05 |
| ACTC1 | -2.21497 | 10.27998 | -6.48711 | 7.80E-08 | 9.34E-07 |
| SCRG1 | -2.1911 | 9.881098 | -8.79016 | 4.42E-11 | 5.36E-09 |
| IFI30 | 2.151372 | 13.55972 | 6.761733 | 3.14E-08 | 4.46E-07 |
| CNN1 | -2.06923 | 10.14173 | -9.00523 | 2.26E-11 | 3.66E-09 |
| RGS5 | -2.05081 | 11.73412 | -8.3158 | 1.99E-10 | 1.30E-08 |
| MRAP2 | -2.03076 | 10.14176 | -7.76925 | 1.15E-09 | 4.14E-08 |
| TCEAL2 | -1.98587 | 9.819182 | -6.37333 | 1.14E-07 | 1.27E-06 |
| SOST | -1.98534 | 9.263892 | -5.42973 | 2.59E-06 | 1.70E-05 |
| CCL18 | 1.977551 | 10.72706 | 5.301283 | 3.95E-06 | 2.47E-05 |
| PLIN2 | 1.953857 | 12.11716 | 6.806408 | 2.71E-08 | 3.99E-07 |
| NPNT | -1.94462 | 9.690377 | -9.46709 | 5.43E-12 | 1.90E-09 |
| CCL19 | 1.922761 | 9.626364 | 4.999783 | 1.06E-05 | 5.87E-05 |
| AQP9 | 1.918837 | 10.43428 | 7.266107 | 5.95E-09 | 1.27E-07 |
| CYTL1 | -1.88581 | 10.38668 | -6.62036 | 5.01E-08 | 6.48E-07 |
| PLA2G7 | 1.879149 | 10.3285 | 6.253312 | 1.69E-07 | 1.76E-06 |
| RASL12 | -1.8649 | 9.700167 | -9.14842 | 1.45E-11 | 3.03E-09 |
| CTSB | 1.849915 | 13.20318 | 7.337072 | 4.72E-09 | 1.08E-07 |
| NOV | -1.84126 | 13.0372 | -9.38199 | 7.04E-12 | 2.12E-09 |
| ACP5 | 1.828724 | 10.49858 | 6.41264 | 9.98E-08 | 1.13E-06 |
| APOC1 | 1.823968 | 11.36811 | 5.184539 | 5.79E-06 | 3.44E-05 |
| TMEM47 | -1.79286 | 11.89566 | -8.37167 | 1.66E-10 | 1.17E-08 |
| UCP2 | 1.781086 | 11.37357 | 7.246727 | 6.35E-09 | 1.34E-07 |
| ACKR1 | 1.772823 | 9.746677 | 5.50801 | 2.00E-06 | 1.37E-05 |
| MARCO | 1.756591 | 10.26377 | 4.951452 | 1.24E-05 | 6.72E-05 |
| FBLN5 | -1.75659 | 9.674662 | -11.3787 | 1.98E-14 | 1.70E-10 |
| KCNA5 | -1.75646 | 9.403886 | -6.67585 | 4.17E-08 | 5.57E-07 |
| SPEG | -1.74992 | 9.546864 | -8.50836 | 1.08E-10 | 9.39E-09 |
| MYH10 | -1.74036 | 13.63888 | -7.7619 | 1.18E-09 | 4.19E-08 |
| FRZB | -1.73624 | 12.56897 | -7.14512 | 8.86E-09 | 1.69E-07 |
| CD36 | 1.734988 | 9.782962 | 7.693521 | 1.47E-09 | 4.97E-08 |
| PPP1R14A | -1.73388 | 11.42498 | -8.10816 | 3.86E-10 | 1.92E-08 |
| SLC2A5 | 1.733747 | 10.4842 | 6.24677 | 1.73E-07 | 1.78E-06 |
| SFRP1 | -1.73261 | 8.667031 | -6.28737 | 1.51E-07 | 1.60E-06 |
| MFAP4 | -1.72296 | 12.40969 | -7.36773 | 4.27E-09 | 1.00E-07 |
| LMOD1 | -1.71767 | 9.49946 | -9.06381 | 1.88E-11 | 3.40E-09 |
| HK3 | 1.705624 | 9.478512 | 8.446237 | 1.31E-10 | 1.03E-08 |
| NEXN | -1.69514 | 10.35547 | -8.48146 | 1.17E-10 | 9.69E-09 |
| CYP1B1 | 1.68613 | 10.12909 | 8.664944 | 6.56E-11 | 6.83E-09 |
| CD163 | 1.684051 | 11.16925 | 7.14714 | 8.80E-09 | 1.69E-07 |
| NPL | 1.680618 | 10.69885 | 7.253149 | 6.21E-09 | 1.32E-07 |
| PPP1R3C | -1.67818 | 11.64193 | -8.08171 | 4.20E-10 | 2.06E-08 |
| HCST | 1.676212 | 12.39525 | 7.560484 | 2.27E-09 | 6.63E-08 |
| FBP1 | 1.663716 | 10.45686 | 6.295164 | 1.47E-07 | 1.57E-06 |
| FGR | 1.650257 | 10.37529 | 8.211375 | 2.77E-10 | 1.56E-08 |
| HAVCR2 | 1.647844 | 12.02866 | 7.516631 | 2.62E-09 | 7.20E-08 |
| SBSPON | -1.64535 | 8.542842 | -7.0413 | 1.25E-08 | 2.18E-07 |
| MYOM1 | -1.64529 | 9.189385 | -7.07572 | 1.11E-08 | 2.01E-07 |
| TTYH3 | 1.634675 | 9.952717 | 9.307559 | 8.86E-12 | 2.33E-09 |
| RGS1 | 1.633228 | 11.08217 | 6.188709 | 2.10E-07 | 2.09E-06 |
| ITGA8 | -1.63213 | 10.34274 | -5.3933 | 2.92E-06 | 1.89E-05 |
| PLEK | 1.626476 | 11.3725 | 6.435552 | 9.25E-08 | 1.07E-06 |
| SNX10 | 1.61604 | 10.80865 | 7.35575 | 4.44E-09 | 1.03E-07 |
| LAPTM5 | 1.599423 | 12.59827 | 6.123317 | 2.61E-07 | 2.47E-06 |
| ITGAM | 1.59799 | 10.57456 | 7.900763 | 7.53E-10 | 3.17E-08 |
| ATP1A2 | -1.59687 | 8.611695 | -6.88367 | 2.10E-08 | 3.26E-07 |
| EFHD1 | -1.59313 | 12.19138 | -7.05846 | 1.18E-08 | 2.10E-07 |
| FLNC | -1.58459 | 9.489973 | -7.21971 | 6.93E-09 | 1.42E-07 |
| CXCL8 | 1.579799 | 9.306258 | 5.821554 | 7.10E-07 | 5.71E-06 |
| DUSP26 | -1.5771 | 8.978754 | -7.66001 | 1.64E-09 | 5.36E-08 |
| CCDC3 | -1.57241 | 11.72792 | -8.70002 | 5.87E-11 | 6.27E-09 |
| SLC25A4 | -1.57236 | 10.24571 | -10.6591 | 1.55E-13 | 3.78E-10 |
| LCP1 | 1.569723 | 12.65107 | 6.978722 | 1.53E-08 | 2.54E-07 |
| CAP2 | -1.56468 | 10.33515 | -8.77508 | 4.64E-11 | 5.54E-09 |
| PARM1 | -1.56187 | 10.00167 | -7.36585 | 4.29E-09 | 1.01E-07 |
| SPARCL1 | -1.55916 | 13.0428 | -7.97815 | 5.87E-10 | 2.67E-08 |
| VAMP8 | 1.558342 | 12.33862 | 7.028687 | 1.30E-08 | 2.24E-07 |
| PTGDS | 1.555023 | 9.416098 | 5.91295 | 5.24E-07 | 4.43E-06 |
| C5AR1 | 1.552436 | 10.11236 | 9.034572 | 2.06E-11 | 3.61E-09 |
| HSPB6 | -1.54825 | 10.57212 | -7.16131 | 8.40E-09 | 1.64E-07 |
| RAC2 | 1.545803 | 11.88589 | 6.6121 | 5.15E-08 | 6.61E-07 |
| VMO1 | 1.541155 | 9.882975 | 5.463307 | 2.32E-06 | 1.55E-05 |
| PLAUR | 1.540439 | 10.17334 | 6.65711 | 4.44E-08 | 5.83E-07 |
| PLN | -1.53852 | 8.805161 | -8.82317 | 3.99E-11 | 5.08E-09 |
| LIPA | 1.536109 | 13.31776 | 7.206047 | 7.25E-09 | 1.47E-07 |
| PRUNE2 | -1.53582 | 10.95495 | -7.44563 | 3.31E-09 | 8.44E-08 |
| CALD1 | -1.52923 | 12.12838 | -5.84361 | 6.60E-07 | 5.38E-06 |
| LDOC1 | -1.52717 | 10.13554 | -6.82241 | 2.57E-08 | 3.82E-07 |
| CA2 | 1.520506 | 9.553683 | 7.550387 | 2.35E-09 | 6.79E-08 |
| APOE | 1.519925 | 14.1434 | 4.245198 | 0.000118 | 0.000483 |
| CD68 | 1.514312 | 13.1617 | 6.757062 | 3.19E-08 | 4.52E-07 |
| SLC14A1 | -1.51418 | 9.564184 | -8.24313 | 2.51E-10 | 1.49E-08 |
| LMCD1 | -1.51396 | 10.97415 | -8.9536 | 2.65E-11 | 3.93E-09 |
| LTBP4 | -1.51277 | 11.29596 | -7.7149 | 1.38E-09 | 4.72E-08 |
| NCKAP1L | 1.511948 | 9.085811 | 9.741262 | 2.36E-12 | 1.13E-09 |
| MYL9 | -1.50856 | 11.715 | -8.17003 | 3.17E-10 | 1.69E-08 |
| CRYAB | -1.50601 | 12.2615 | -8.17985 | 3.07E-10 | 1.66E-08 |
| ANPEP | 1.504146 | 9.652491 | 7.344311 | 4.61E-09 | 1.06E-07 |
| ADAM8 | 1.502568 | 9.400583 | 7.168355 | 8.21E-09 | 1.61E-07 |
| ANKRD33 | 1.50105 | 11.30904 | 7.619122 | 1.88E-09 | 5.74E-08 |
| PDLIM3 | -1.5003 | 11.2872 | -10.0199 | 1.02E-12 | 7.83E-10 |
| CFD | 1.496331 | 11.90441 | 5.036808 | 9.38E-06 | 5.25E-05 |
| MYH11 | -1.49599 | 14.17731 | -7.56493 | 2.24E-09 | 6.56E-08 |
| COL21A1 | -1.49386 | 10.47049 | -8.65791 | 6.71E-11 | 6.90E-09 |
| MYLK | -1.49114 | 10.78244 | -8.62122 | 7.53E-11 | 7.53E-09 |
| MAPK13 | 1.488883 | 9.402215 | 7.620223 | 1.87E-09 | 5.73E-08 |
| ANO1 | -1.48583 | 9.504008 | -10.5555 | 2.09E-13 | 3.90E-10 |
| CARTPT | -1.47824 | 7.940775 | -4.94978 | 1.24E-05 | 6.75E-05 |
| SGK1 | 1.473525 | 12.39269 | 7.045617 | 1.23E-08 | 2.16E-07 |
| PLBD1 | 1.471658 | 11.43425 | 6.800092 | 2.77E-08 | 4.03E-07 |
| STXBP2 | 1.471176 | 10.15464 | 8.626641 | 7.40E-11 | 7.44E-09 |
| TYROBP | 1.470435 | 13.75942 | 6.802897 | 2.74E-08 | 4.02E-07 |
| CSRP2 | -1.46894 | 10.16374 | -7.42459 | 3.54E-09 | 8.89E-08 |
| FMOD | -1.46223 | 10.57055 | -9.41793 | 6.31E-12 | 2.05E-09 |
| CASQ2 | -1.45837 | 8.461234 | -5.83344 | 6.82E-07 | 5.52E-06 |
| YAP1 | -1.45552 | 11.49522 | -8.55371 | 9.32E-11 | 8.66E-09 |
| PGD | 1.454172 | 10.77866 | 7.798254 | 1.05E-09 | 3.90E-08 |
| LIMCH1 | -1.45202 | 10.33282 | -9.76143 | 2.22E-12 | 1.12E-09 |
| SMTN | -1.45155 | 9.231586 | -7.6211 | 1.87E-09 | 5.73E-08 |
| ADM | 1.447118 | 10.38304 | 6.292014 | 1.49E-07 | 1.59E-06 |
| CD14 | 1.441674 | 13.1332 | 5.493475 | 2.10E-06 | 1.42E-05 |
| SCD | 1.440539 | 11.91429 | 5.088736 | 7.92E-06 | 4.52E-05 |
| ASPN | -1.43848 | 12.20156 | -7.61395 | 1.91E-09 | 5.80E-08 |
| SHC4 | -1.43459 | 9.16541 | -7.93429 | 6.76E-10 | 2.96E-08 |
| EMILIN2 | 1.434197 | 11.10625 | 6.459391 | 8.55E-08 | 1.00E-06 |
| PLS3 | -1.43314 | 11.33224 | -10.0106 | 1.05E-12 | 7.83E-10 |
| CCL3 | 1.432434 | 11.076 | 5.344581 | 3.43E-06 | 2.18E-05 |
| FCER1G | 1.431287 | 13.20105 | 5.587324 | 1.54E-06 | 1.09E-05 |
| CD300A | 1.429212 | 9.876735 | 7.04371 | 1.24E-08 | 2.17E-07 |
| FOXC1 | -1.42629 | 10.99183 | -6.79888 | 2.78E-08 | 4.04E-07 |
| CPVL | 1.41925 | 11.00575 | 5.898839 | 5.49E-07 | 4.59E-06 |
| LILRB3 | 1.416961 | 10.42685 | 7.273135 | 5.82E-09 | 1.25E-07 |
| MYLIP | -1.41022 | 11.5743 | -9.70079 | 2.66E-12 | 1.19E-09 |
| FHL5 | -1.40694 | 8.54107 | -6.95566 | 1.65E-08 | 2.68E-07 |
| HS3ST2 | 1.401939 | 10.26649 | 5.467764 | 2.28E-06 | 1.53E-05 |
| CPED1 | -1.39867 | 10.68996 | -6.8052 | 2.72E-08 | 4.00E-07 |
| RNASE1 | 1.398444 | 13.7861 | 5.354423 | 3.32E-06 | 2.12E-05 |
| LHFPL2 | 1.394958 | 11.196 | 8.526219 | 1.02E-10 | 9.06E-09 |
| FHL1 | -1.39038 | 12.6957 | -7.95181 | 6.39E-10 | 2.86E-08 |
| TNFRSF21 | 1.388022 | 10.51646 | 7.767724 | 1.16E-09 | 4.15E-08 |
| REEP1 | -1.38659 | 8.426377 | -7.62166 | 1.86E-09 | 5.73E-08 |
| ADAP2 | 1.382301 | 11.17427 | 7.624729 | 1.84E-09 | 5.70E-08 |
| KCNMB1 | -1.38217 | 10.64214 | -9.54189 | 4.32E-12 | 1.58E-09 |
| IGFBP6 | -1.37959 | 12.12899 | -9.05284 | 1.95E-11 | 3.49E-09 |
| NOX4 | -1.37031 | 10.00533 | -7.54736 | 2.37E-09 | 6.82E-08 |
| CCDC146 | -1.36964 | 8.458878 | -8.35467 | 1.75E-10 | 1.21E-08 |
| SLC16A10 | 1.369004 | 9.21129 | 7.457166 | 3.19E-09 | 8.18E-08 |
| COL4A5 | -1.36877 | 9.604808 | -9.62697 | 3.33E-12 | 1.36E-09 |
| TMEM119 | 1.368523 | 9.466975 | 6.307077 | 1.42E-07 | 1.52E-06 |
| DACT3 | -1.36661 | 8.827101 | -8.11045 | 3.83E-10 | 1.92E-08 |
| HSPB8 | -1.36276 | 9.306194 | -7.15729 | 8.51E-09 | 1.65E-07 |
| CCR1 | 1.360094 | 9.13327 | 9.382207 | 7.04E-12 | 2.12E-09 |
| RAMP1 | -1.35906 | 8.719757 | -7.61018 | 1.93E-09 | 5.85E-08 |
| GEM | -1.35791 | 9.059134 | -10.0889 | 8.30E-13 | 7.35E-10 |
| LGMN | 1.349743 | 13.37916 | 6.27042 | 1.60E-07 | 1.68E-06 |
| ADAMTS8 | -1.34709 | 9.741621 | -5.66683 | 1.18E-06 | 8.78E-06 |
| MAFB | 1.346325 | 12.10287 | 6.736534 | 3.41E-08 | 4.76E-07 |
| SLC15A3 | 1.345532 | 11.50508 | 6.671969 | 4.23E-08 | 5.61E-07 |
| AOC3 | -1.34257 | 8.874759 | -8.85043 | 3.66E-11 | 4.84E-09 |
| GREM1 | 1.340949 | 8.48034 | 7.829041 | 9.50E-10 | 3.68E-08 |
| FPR3 | 1.340864 | 10.98303 | 5.856631 | 6.32E-07 | 5.19E-06 |
| SPI1 | 1.339375 | 10.36084 | 8.395578 | 1.54E-10 | 1.13E-08 |
| OMD | -1.33843 | 10.58776 | -7.87682 | 8.14E-10 | 3.30E-08 |
| C2 | 1.335051 | 9.575071 | 6.797557 | 2.79E-08 | 4.05E-07 |
| SMOC2 | -1.33351 | 11.45492 | -7.46556 | 3.10E-09 | 8.02E-08 |
| CRIM1 | -1.32768 | 8.563488 | -8.93152 | 2.84E-11 | 4.08E-09 |
| MYO1G | 1.327204 | 9.849823 | 6.474955 | 8.12E-08 | 9.59E-07 |
| C1QTNF7 | -1.32124 | 8.393627 | -10.2298 | 5.46E-13 | 6.25E-10 |
| SLA | 1.319238 | 10.55358 | 7.780415 | 1.11E-09 | 4.04E-08 |
| HACD1 | -1.31585 | 9.040122 | -7.69223 | 1.48E-09 | 4.98E-08 |
| EPB41L3 | 1.315837 | 11.58356 | 6.711019 | 3.71E-08 | 5.09E-07 |
| LYN | 1.314332 | 11.29658 | 6.675006 | 4.18E-08 | 5.58E-07 |
| MTURN | -1.31103 | 9.814937 | -8.53179 | 1.00E-10 | 8.95E-09 |
| C1QC | 1.310874 | 13.26271 | 5.113105 | 7.31E-06 | 4.22E-05 |
| LY86 | 1.307136 | 10.68928 | 7.068851 | 1.14E-08 | 2.04E-07 |
| SGCA | -1.30581 | 9.735419 | -7.20543 | 7.27E-09 | 1.47E-07 |
| WFS1 | -1.30426 | 10.14022 | -8.3359 | 1.86E-10 | 1.24E-08 |
| SLC2A3 | 1.303576 | 11.39169 | 6.628399 | 4.88E-08 | 6.34E-07 |
| WAS | 1.303101 | 11.09939 | 7.639958 | 1.75E-09 | 5.55E-08 |
| C1QA | 1.301552 | 13.73017 | 5.665104 | 1.19E-06 | 8.81E-06 |
| LPXN | 1.300666 | 11.30714 | 8.217125 | 2.72E-10 | 1.56E-08 |
| FERMT2 | -1.29754 | 11.773 | -9.76157 | 2.22E-12 | 1.12E-09 |
| CTSS | 1.29418 | 9.837037 | 7.498521 | 2.78E-09 | 7.49E-08 |
| PAM | -1.29281 | 11.8089 | -10.6151 | 1.76E-13 | 3.78E-10 |
| GRN | 1.291652 | 11.72445 | 7.604806 | 1.97E-09 | 5.90E-08 |
| SLAMF8 | 1.290775 | 9.885323 | 7.201554 | 7.36E-09 | 1.49E-07 |
| CYP27A1 | 1.290381 | 11.71792 | 6.034466 | 3.50E-07 | 3.17E-06 |
| C1orf162 | 1.288713 | 12.04784 | 6.47858 | 8.02E-08 | 9.52E-07 |
| KYNU | 1.288291 | 9.650534 | 7.691465 | 1.48E-09 | 4.98E-08 |
| NR1H3 | 1.288041 | 10.17143 | 7.38593 | 4.02E-09 | 9.61E-08 |
| CYTH4 | 1.28542 | 10.72536 | 6.959317 | 1.63E-08 | 2.66E-07 |
| SPP1 | 1.285166 | 14.81211 | 4.018758 | 0.000237 | 0.000896 |
| GAL | 1.283406 | 8.416679 | 4.861364 | 1.66E-05 | 8.67E-05 |
| CYBA | 1.282043 | 12.89134 | 6.545571 | 6.42E-08 | 7.92E-07 |
| FCN1 | 1.278747 | 9.971835 | 5.459369 | 2.35E-06 | 1.57E-05 |
| MRGPRF | -1.27813 | 9.846373 | -7.63312 | 1.79E-09 | 5.60E-08 |
| GPSM3 | 1.277928 | 10.70563 | 7.373625 | 4.19E-09 | 9.91E-08 |
| CCL8 | 1.277178 | 11.29831 | 4.350875 | 8.44E-05 | 0.000362 |
| GJA1 | -1.27411 | 11.32309 | -7.48271 | 2.93E-09 | 7.75E-08 |
| FCGRT | 1.272733 | 12.08828 | 7.520381 | 2.59E-09 | 7.15E-08 |
| CD84 | 1.271896 | 9.542145 | 6.463764 | 8.43E-08 | 9.90E-07 |
| CD53 | 1.271603 | 10.0863 | 6.938811 | 1.75E-08 | 2.80E-07 |
| ITGA10 | -1.27115 | 9.317203 | -8.00226 | 5.43E-10 | 2.52E-08 |
| PCDH20 | -1.2701 | 7.873856 | -6.36868 | 1.16E-07 | 1.28E-06 |
| CTSG | 1.26879 | 9.246988 | 4.25982 | 0.000112 | 0.000465 |
| CXCL16 | 1.263762 | 12.38804 | 6.5263 | 6.85E-08 | 8.33E-07 |
| PTGIS | -1.26266 | 11.63428 | -9.02538 | 2.12E-11 | 3.66E-09 |
| SCARA3 | -1.26175 | 8.623285 | -7.10837 | 1.00E-08 | 1.84E-07 |
| SSPN | -1.25882 | 10.07997 | -6.95222 | 1.67E-08 | 2.70E-07 |
| CSTB | 1.249913 | 14.13558 | 7.164349 | 8.32E-09 | 1.63E-07 |
| SDS | 1.249892 | 8.917607 | 5.701757 | 1.05E-06 | 7.97E-06 |
| FUCA1 | 1.242889 | 12.41937 | 6.315529 | 1.38E-07 | 1.49E-06 |
| LPP | -1.23718 | 13.07371 | -7.98369 | 5.76E-10 | 2.65E-08 |
| APBB1IP | 1.235974 | 9.779416 | 8.2287 | 2.62E-10 | 1.52E-08 |
| ECM2 | -1.23407 | 8.898226 | -8.30067 | 2.08E-10 | 1.35E-08 |
| CD37 | 1.232041 | 10.66495 | 5.630089 | 1.34E-06 | 9.74E-06 |
| IGSF6 | 1.228156 | 11.56592 | 4.516546 | 5.00E-05 | 0.000228 |
| CSRP1 | -1.22336 | 12.91634 | -9.22105 | 1.16E-11 | 2.66E-09 |
| HCK | 1.221399 | 9.979642 | 7.591631 | 2.05E-09 | 6.11E-08 |
| MAPKAPK3 | 1.221199 | 10.4771 | 9.09193 | 1.72E-11 | 3.20E-09 |
| SGCE | -1.22053 | 10.31864 | -8.43103 | 1.38E-10 | 1.06E-08 |
| GNA15 | 1.217722 | 10.00289 | 7.034804 | 1.27E-08 | 2.21E-07 |
| ITGB2 | 1.217184 | 13.80901 | 5.571472 | 1.62E-06 | 1.15E-05 |
| RGCC | 1.214697 | 12.41933 | 6.59608 | 5.43E-08 | 6.90E-07 |
| BCAT1 | 1.214432 | 11.20397 | 6.155932 | 2.34E-07 | 2.29E-06 |
| NRROS | 1.21407 | 9.343102 | 9.008994 | 2.23E-11 | 3.66E-09 |
| PYCARD | 1.213668 | 10.93751 | 7.554559 | 2.32E-09 | 6.72E-08 |
| CTSZ | 1.210607 | 13.0622 | 6.82718 | 2.53E-08 | 3.78E-07 |
| TNFRSF1B | 1.210497 | 10.67041 | 6.827235 | 2.53E-08 | 3.78E-07 |
| LEFTY2 | -1.21009 | 8.131452 | -7.41479 | 3.66E-09 | 9.04E-08 |
| IRF8 | 1.210003 | 10.6732 | 5.878255 | 5.88E-07 | 4.88E-06 |
| LAYN | -1.20645 | 8.515113 | -9.87889 | 1.56E-12 | 9.83E-10 |
| C15orf48 | 1.205243 | 8.773196 | 6.883 | 2.10E-08 | 3.27E-07 |
| SLC39A8 | 1.205201 | 9.209928 | 7.162334 | 8.37E-09 | 1.64E-07 |
| PKD2 | -1.20515 | 10.09996 | -7.54434 | 2.40E-09 | 6.85E-08 |
| ABCA1 | 1.20369 | 11.78477 | 4.684712 | 2.93E-05 | 0.000142 |
| CPE | -1.20336 | 11.10045 | -5.44259 | 2.48E-06 | 1.64E-05 |
| SLC24A3 | -1.2033 | 9.590812 | -7.18948 | 7.66E-09 | 1.53E-07 |
| HEY2 | -1.20213 | 8.688151 | -8.7026 | 5.83E-11 | 6.27E-09 |
| SLAMF9 | 1.201811 | 8.325971 | 6.57873 | 5.76E-08 | 7.22E-07 |
| CD83 | 1.201578 | 10.12451 | 6.692752 | 3.94E-08 | 5.33E-07 |
| SLC31A2 | 1.200879 | 10.49053 | 6.677622 | 4.15E-08 | 5.55E-07 |
| DOCK2 | 1.200073 | 10.66703 | 7.036096 | 1.27E-08 | 2.20E-07 |
| IL1RN | 1.199111 | 8.686473 | 6.381533 | 1.11E-07 | 1.24E-06 |
| FILIP1L | -1.19524 | 9.642977 | -10.9183 | 7.32E-14 | 3.63E-10 |
| GPR183 | 1.19452 | 9.755592 | 6.709692 | 3.73E-08 | 5.11E-07 |
| LPL | 1.193823 | 9.379055 | 5.30005 | 3.97E-06 | 2.47E-05 |
| MTMR11 | -1.19351 | 10.25563 | -8.70069 | 5.86E-11 | 6.27E-09 |
| TPP1 | 1.192228 | 12.06944 | 6.73191 | 3.47E-08 | 4.82E-07 |
| CDH13 | -1.19193 | 10.6258 | -8.9548 | 2.64E-11 | 3.93E-09 |
| ADAMTS1 | -1.18944 | 10.29068 | -6.90493 | 1.96E-08 | 3.08E-07 |
| TCN2 | 1.188666 | 9.885338 | 6.493996 | 7.62E-08 | 9.17E-07 |
| NPTX2 | -1.18679 | 9.399913 | -6.08736 | 2.94E-07 | 2.74E-06 |
| GLA | 1.186742 | 11.03203 | 8.422031 | 1.42E-10 | 1.06E-08 |
| RNASET2 | 1.186171 | 13.14337 | 7.139001 | 9.04E-09 | 1.72E-07 |
| PRRT2 | -1.18483 | 8.680419 | -7.55663 | 2.30E-09 | 6.69E-08 |
| FAM180A | -1.17969 | 9.388113 | -9.20632 | 1.21E-11 | 2.74E-09 |
| CNDP2 | 1.178546 | 12.37462 | 8.023811 | 5.06E-10 | 2.41E-08 |
| LIMS2 | -1.17673 | 9.552069 | -8.85523 | 3.61E-11 | 4.84E-09 |
| MYO1D | -1.17484 | 10.83619 | -8.20828 | 2.80E-10 | 1.57E-08 |
| SHROOM3 | -1.17472 | 8.35056 | -7.53544 | 2.47E-09 | 6.93E-08 |
| CSF1R | 1.174283 | 12.71124 | 5.966152 | 4.39E-07 | 3.80E-06 |
| ACTG2 | -1.17427 | 12.60475 | -5.3617 | 3.24E-06 | 2.08E-05 |
| RENBP | 1.172816 | 10.38798 | 7.560001 | 2.28E-09 | 6.63E-08 |
| NABP1 | 1.171795 | 10.62985 | 8.290751 | 2.15E-10 | 1.37E-08 |
| PLEKHO2 | 1.171737 | 10.0053 | 8.344276 | 1.81E-10 | 1.23E-08 |
| GM2A | 1.168577 | 12.28854 | 6.593584 | 5.48E-08 | 6.94E-07 |
| KANK2 | -1.16849 | 10.7694 | -7.40721 | 3.75E-09 | 9.19E-08 |
| AIF1L | -1.1684 | 10.64944 | -4.57976 | 4.09E-05 | 0.000191 |
| SLMAP | -1.16759 | 10.81766 | -6.98941 | 1.48E-08 | 2.47E-07 |
| OLR1 | 1.16573 | 10.84619 | 4.233959 | 0.000122 | 0.000499 |
| DYSF | 1.165382 | 9.47521 | 6.615522 | 5.09E-08 | 6.55E-07 |
| FOLR2 | 1.16273 | 10.7487 | 5.859486 | 6.26E-07 | 5.15E-06 |
| LFNG | 1.162053 | 10.01257 | 6.547053 | 6.39E-08 | 7.90E-07 |
| FIBIN | -1.16169 | 8.431914 | -7.53642 | 2.46E-09 | 6.93E-08 |
| BAMBI | -1.15948 | 9.344451 | -6.44132 | 9.08E-08 | 1.05E-06 |
| EPSTI1 | 1.15803 | 9.877651 | 6.616392 | 5.08E-08 | 6.54E-07 |
| CD4 | 1.155532 | 9.471129 | 7.458388 | 3.17E-09 | 8.18E-08 |
| IBSP | 1.154948 | 8.519924 | 5.772696 | 8.34E-07 | 6.55E-06 |
| CD48 | 1.154437 | 9.433258 | 6.337219 | 1.28E-07 | 1.40E-06 |
| TMEM51 | 1.154272 | 10.91883 | 7.182294 | 7.84E-09 | 1.56E-07 |
| CAV2 | -1.15404 | 10.15439 | -9.2198 | 1.16E-11 | 2.66E-09 |
| GMIP | 1.152051 | 9.398193 | 10.09287 | 8.20E-13 | 7.35E-10 |
| MPP1 | 1.151989 | 10.56688 | 7.046005 | 1.23E-08 | 2.16E-07 |
| ZNF385A | 1.150397 | 8.807609 | 8.272569 | 2.28E-10 | 1.41E-08 |
| NFIX | -1.14964 | 11.49859 | -6.9702 | 1.58E-08 | 2.59E-07 |
| ATOH8 | -1.14964 | 8.599727 | -6.7682 | 3.07E-08 | 4.39E-07 |
| TPSB2 | 1.14707 | 10.59022 | 2.852575 | 0.006693 | 0.017464 |
| PHGDH | -1.14403 | 9.59005 | -6.7038 | 3.80E-08 | 5.17E-07 |
| FERMT3 | 1.142818 | 9.554711 | 7.764172 | 1.17E-09 | 4.18E-08 |
| SAT1 | 1.142244 | 13.29839 | 7.105133 | 1.01E-08 | 1.86E-07 |
| NCALD | -1.13986 | 9.381742 | -8.78743 | 4.46E-11 | 5.36E-09 |
| CRIP2 | -1.13904 | 11.84161 | -7.00551 | 1.40E-08 | 2.36E-07 |
| HAMP | 1.137722 | 9.166082 | 5.276341 | 4.29E-06 | 2.64E-05 |
| KLHL42 | -1.13698 | 10.2507 | -8.81958 | 4.03E-11 | 5.09E-09 |
| DRAM1 | 1.136696 | 11.00569 | 6.541507 | 6.51E-08 | 8.01E-07 |
| EXT1 | -1.13642 | 10.28227 | -8.47351 | 1.20E-10 | 9.75E-09 |
| PID1 | -1.13438 | 8.918621 | -8.55634 | 9.25E-11 | 8.64E-09 |
| HSPA2 | -1.13315 | 9.02488 | -10.1706 | 6.51E-13 | 6.58E-10 |
| LAMA5 | -1.13291 | 11.1708 | -7.25523 | 6.17E-09 | 1.31E-07 |
| CHST13 | 1.129959 | 8.716156 | 7.900572 | 7.54E-10 | 3.17E-08 |
| RERG | -1.12965 | 9.39799 | -7.53193 | 2.50E-09 | 6.95E-08 |
| C1QB | 1.12955 | 13.74302 | 4.963997 | 1.19E-05 | 6.50E-05 |
| SCARB1 | 1.128773 | 10.437 | 6.137095 | 2.49E-07 | 2.40E-06 |
| DCSTAMP | 1.126825 | 8.417755 | 8.567445 | 8.93E-11 | 8.47E-09 |
| TBXAS1 | 1.126232 | 10.55603 | 7.196467 | 7.49E-09 | 1.50E-07 |
| SLC1A3 | 1.125256 | 10.47675 | 5.324187 | 3.66E-06 | 2.31E-05 |
| FLNA | -1.12451 | 10.92811 | -7.40367 | 3.79E-09 | 9.26E-08 |
| TCEA3 | -1.12323 | 10.38253 | -8.0132 | 5.24E-10 | 2.45E-08 |
| HK2 | 1.121878 | 9.332857 | 4.562076 | 4.33E-05 | 0.000201 |
| SERPINF1 | 1.121438 | 9.651201 | 5.766241 | 8.52E-07 | 6.67E-06 |
| HLA-DRB3 | 1.121293 | 12.23702 | 3.776104 | 0.000493 | 0.001721 |
| WFDC1 | -1.11966 | 9.246335 | -6.62414 | 4.95E-08 | 6.41E-07 |
| ACP2 | 1.119479 | 9.197171 | 8.386421 | 1.59E-10 | 1.15E-08 |
| NCEH1 | 1.118592 | 9.449677 | 7.607579 | 1.95E-09 | 5.87E-08 |
| MCUB | 1.117328 | 10.2854 | 7.720253 | 1.35E-09 | 4.67E-08 |
| ATF5 | 1.115206 | 10.49786 | 6.338228 | 1.28E-07 | 1.40E-06 |
| NXPH3 | -1.11444 | 8.40433 | -9.16099 | 1.39E-11 | 3.01E-09 |
| S100A8 | 1.114411 | 9.947739 | 4.853082 | 1.70E-05 | 8.87E-05 |
| MMD | 1.113118 | 10.17942 | 8.108171 | 3.86E-10 | 1.92E-08 |
| CTSL | 1.112325 | 11.12015 | 6.158127 | 2.32E-07 | 2.27E-06 |
| MGAT1 | 1.112073 | 11.6631 | 9.258526 | 1.03E-11 | 2.49E-09 |
| SORBS1 | -1.11188 | 9.122486 | -8.88825 | 3.25E-11 | 4.50E-09 |
| SPARC | -1.10972 | 13.63124 | -8.21529 | 2.74E-10 | 1.56E-08 |
| HEYL | -1.10474 | 10.14482 | -7.78135 | 1.11E-09 | 4.04E-08 |
| COL14A1 | -1.103 | 9.369746 | -7.88362 | 7.96E-10 | 3.29E-08 |
| CRISPLD1 | -1.10191 | 8.448105 | -6.59501 | 5.45E-08 | 6.92E-07 |
| AIF1 | 1.101478 | 11.58216 | 6.269402 | 1.61E-07 | 1.68E-06 |
| HCLS1 | 1.101409 | 11.67678 | 5.85043 | 6.45E-07 | 5.27E-06 |
| SIRPA | 1.100549 | 12.59444 | 6.674077 | 4.20E-08 | 5.59E-07 |
| BTK | 1.100161 | 9.970382 | 7.360662 | 4.37E-09 | 1.02E-07 |
| LCP2 | 1.095675 | 10.85456 | 5.695235 | 1.08E-06 | 8.11E-06 |
| CTSD | 1.094241 | 14.11734 | 7.459199 | 3.16E-09 | 8.18E-08 |
| GMFG | 1.094221 | 10.05559 | 6.882327 | 2.11E-08 | 3.27E-07 |
| SLC16A3 | 1.093901 | 10.44366 | 7.000829 | 1.43E-08 | 2.39E-07 |
| MSRB3 | -1.09347 | 9.616468 | -10.4652 | 2.73E-13 | 3.90E-10 |
| MFSD1 | 1.092255 | 11.75104 | 7.470401 | 3.05E-09 | 7.96E-08 |
| PILRA | 1.091141 | 9.226451 | 7.476441 | 2.99E-09 | 7.86E-08 |
| VASN | -1.09005 | 11.02644 | -7.21586 | 7.02E-09 | 1.43E-07 |
| TREM2 | 1.090038 | 8.952456 | 7.508471 | 2.69E-09 | 7.34E-08 |
| PLA2G15 | 1.089754 | 9.709336 | 8.806707 | 4.20E-11 | 5.19E-09 |
| FYCO1 | -1.08951 | 10.35191 | -9.39745 | 6.72E-12 | 2.11E-09 |
| CD33 | 1.089327 | 9.672693 | 7.414273 | 3.66E-09 | 9.04E-08 |
| TRPV2 | 1.089206 | 9.858877 | 8.434084 | 1.36E-10 | 1.06E-08 |
| CKMT2 | -1.08792 | 8.445494 | -7.51039 | 2.68E-09 | 7.32E-08 |
| CORO1A | 1.087713 | 10.76721 | 5.974414 | 4.27E-07 | 3.72E-06 |
| TMC6 | 1.086952 | 9.231205 | 7.934439 | 6.76E-10 | 2.96E-08 |
| KCTD10 | -1.08364 | 10.2057 | -6.90263 | 1.97E-08 | 3.10E-07 |
| EFHD2 | 1.082449 | 11.67315 | 7.54067 | 2.43E-09 | 6.88E-08 |
| CEBPA | 1.081185 | 10.33246 | 5.037096 | 9.37E-06 | 5.25E-05 |
| CPXM2 | -1.07918 | 11.59542 | -8.0225 | 5.09E-10 | 2.41E-08 |
| NCKAP1 | -1.07882 | 11.85467 | -7.87268 | 8.25E-10 | 3.33E-08 |
| TYMP | 1.078681 | 9.275795 | 6.382309 | 1.10E-07 | 1.24E-06 |
| PDE8B | -1.07851 | 8.128816 | -6.09268 | 2.89E-07 | 2.70E-06 |
| CSPG4 | -1.07842 | 9.17429 | -8.31536 | 1.99E-10 | 1.30E-08 |
| SEL1L3 | 1.076785 | 10.06658 | 5.748622 | 9.03E-07 | 6.99E-06 |
| CSK | 1.076425 | 11.09044 | 7.158484 | 8.48E-09 | 1.65E-07 |
| MKX | -1.07619 | 8.890961 | -6.6959 | 3.90E-08 | 5.29E-07 |
| NRIP3 | 1.07599 | 9.51381 | 6.738781 | 3.39E-08 | 4.73E-07 |
| PLSCR4 | -1.0748 | 10.71014 | -7.27972 | 5.69E-09 | 1.24E-07 |
| SRGN | 1.074238 | 13.15267 | 5.699994 | 1.06E-06 | 8.01E-06 |
| MAOB | -1.07138 | 8.77385 | -7.97578 | 5.91E-10 | 2.69E-08 |
| IL1B | 1.0708 | 9.487366 | 4.800207 | 2.02E-05 | 0.000103 |
| PDGFD | -1.07076 | 10.06807 | -6.49225 | 7.67E-08 | 9.22E-07 |
| SLC25A23 | -1.07001 | 9.869843 | -7.65161 | 1.69E-09 | 5.42E-08 |
| COLEC12 | 1.069984 | 11.43562 | 5.844417 | 6.58E-07 | 5.36E-06 |
| RASL11B | -1.06905 | 8.904368 | -7.96785 | 6.07E-10 | 2.74E-08 |
| TMEM30B | -1.06788 | 9.222101 | -8.94181 | 2.75E-11 | 4.01E-09 |
| CMTM7 | 1.067311 | 10.01613 | 7.005719 | 1.40E-08 | 2.36E-07 |
| TDO2 | 1.066765 | 8.350873 | 5.536241 | 1.82E-06 | 1.26E-05 |
| ID3 | -1.06652 | 11.45287 | -7.07787 | 1.11E-08 | 2.00E-07 |
| COL16A1 | -1.06636 | 9.611409 | -7.63271 | 1.80E-09 | 5.60E-08 |
| OAF | 1.065385 | 9.285918 | 7.694931 | 1.47E-09 | 4.95E-08 |
| ITGA11 | -1.06491 | 9.933096 | -7.17578 | 8.01E-09 | 1.58E-07 |
| SLCO2A1 | 1.063296 | 9.829624 | 3.697768 | 0.000623 | 0.002119 |
| SLCO2B1 | 1.061647 | 11.14341 | 5.424968 | 2.63E-06 | 1.73E-05 |
| PALLD | -1.05945 | 12.09464 | -7.05935 | 1.18E-08 | 2.09E-07 |
| MT1G | 1.058175 | 9.145934 | 4.042868 | 0.00022 | 0.000839 |
| OSCAR | 1.057772 | 9.085775 | 5.881151 | 5.82E-07 | 4.83E-06 |
| DACT1 | -1.05768 | 9.758112 | -7.33239 | 4.79E-09 | 1.09E-07 |
| TMIGD3 | 1.057146 | 9.498014 | 5.324934 | 3.65E-06 | 2.30E-05 |
| CLDN23 | 1.05627 | 9.300959 | 7.109947 | 9.95E-09 | 1.84E-07 |
| MS4A6A | 1.054156 | 12.23475 | 5.228038 | 5.02E-06 | 3.04E-05 |
| ADRA1B | -1.05205 | 8.108936 | -7.01026 | 1.38E-08 | 2.34E-07 |
| CCL13 | 1.049019 | 9.619192 | 2.982221 | 0.004741 | 0.012875 |
| TNFSF13B | 1.046976 | 10.1307 | 5.901802 | 5.44E-07 | 4.55E-06 |
| IL10RA | 1.044911 | 9.662706 | 6.797367 | 2.79E-08 | 4.05E-07 |
| PARVG | 1.044502 | 9.660739 | 6.752129 | 3.24E-08 | 4.58E-07 |
| CYFIP2 | -1.04397 | 10.58228 | -6.87249 | 2.18E-08 | 3.35E-07 |
| EVI2B | 1.043355 | 10.20001 | 4.886678 | 1.53E-05 | 8.07E-05 |
| MCOLN1 | 1.043263 | 10.13993 | 7.649895 | 1.70E-09 | 5.44E-08 |
| KCNK17 | -1.04274 | 8.210683 | -5.28765 | 4.13E-06 | 2.56E-05 |
| CYBB | 1.041768 | 9.883313 | 6.444308 | 8.99E-08 | 1.05E-06 |
| CHSY3 | -1.04101 | 9.903224 | -8.463 | 1.24E-10 | 9.89E-09 |
| ADIRF | -1.04034 | 13.17331 | -5.58079 | 1.57E-06 | 1.12E-05 |
| CD209 | 1.040309 | 9.731093 | 4.265503 | 0.00011 | 0.000457 |
| GPR137B | 1.039815 | 11.42031 | 6.978894 | 1.53E-08 | 2.54E-07 |
| GAS6 | -1.03965 | 11.38446 | -6.07349 | 3.08E-07 | 2.85E-06 |
| MFSD12 | 1.039238 | 8.970382 | 8.451451 | 1.29E-10 | 1.02E-08 |
| VNN1 | 1.039199 | 9.202173 | 6.959422 | 1.63E-08 | 2.66E-07 |
| CST6 | -1.03917 | 8.928504 | -5.71864 | 9.98E-07 | 7.59E-06 |
| SPINT2 | -1.03897 | 11.3789 | -8.50845 | 1.08E-10 | 9.39E-09 |
| IRAK1 | 1.037405 | 10.98645 | 8.538215 | 9.79E-11 | 8.86E-09 |
| LY96 | 1.03538 | 10.04744 | 8.463381 | 1.24E-10 | 9.89E-09 |
| ME2 | 1.035044 | 10.26142 | 5.471473 | 2.26E-06 | 1.52E-05 |
| CAMK2G | -1.03208 | 9.476317 | -8.43043 | 1.38E-10 | 1.06E-08 |
| LHFP | -1.03173 | 11.189 | -7.94478 | 6.53E-10 | 2.91E-08 |
| GSTM3 | -1.03122 | 9.21677 | -8.25425 | 2.42E-10 | 1.46E-08 |
| HLA-DMA | 1.03086 | 13.04878 | 5.218639 | 5.18E-06 | 3.12E-05 |
| CXCL10 | 1.030394 | 9.337509 | 5.342399 | 3.45E-06 | 2.19E-05 |
| HLA-B | 1.030219 | 13.50917 | 4.663833 | 3.13E-05 | 0.000151 |
| LTBP2 | -1.02964 | 11.03416 | -7.0827 | 1.09E-08 | 1.97E-07 |
| CYGB | 1.028978 | 8.975798 | 7.056954 | 1.18E-08 | 2.10E-07 |
| C5orf46 | -1.02618 | 9.990409 | -6.55321 | 6.26E-08 | 7.76E-07 |
| TMEM130 | -1.02584 | 9.616178 | -5.97518 | 4.26E-07 | 3.72E-06 |
| SQSTM1 | 1.024517 | 10.29345 | 6.971696 | 1.57E-08 | 2.58E-07 |
| C3orf70 | -1.02442 | 8.283653 | -7.95161 | 6.39E-10 | 2.86E-08 |
| VSIG4 | 1.024273 | 10.40358 | 4.909169 | 1.42E-05 | 7.56E-05 |
| LGALS9 | 1.023203 | 8.598547 | 8.501577 | 1.10E-10 | 9.49E-09 |
| DSTN | -1.02298 | 10.92045 | -7.13137 | 9.27E-09 | 1.75E-07 |
| HLA-DMB | 1.021546 | 12.97418 | 4.657221 | 3.20E-05 | 0.000153 |
| PLAU | 1.019876 | 11.26814 | 5.678125 | 1.14E-06 | 8.49E-06 |
| CAV1 | -1.01791 | 9.274855 | -6.00128 | 3.91E-07 | 3.47E-06 |
| CAPG | 1.016538 | 9.757149 | 5.791704 | 7.83E-07 | 6.21E-06 |
| ALDH1B1 | -1.01588 | 8.369303 | -6.81577 | 2.63E-08 | 3.89E-07 |
| CTSK | 1.015642 | 12.40417 | 5.497631 | 2.07E-06 | 1.41E-05 |
| JAM3 | -1.01556 | 11.43804 | -6.44221 | 9.05E-08 | 1.05E-06 |
| PLTP | 1.014788 | 11.39896 | 3.774551 | 0.000496 | 0.001729 |
| SLC22A3 | -1.01473 | 8.146535 | -6.8057 | 2.71E-08 | 3.99E-07 |
| SDCBP | 1.010707 | 11.61201 | 6.74812 | 3.28E-08 | 4.61E-07 |
| ZNF385D | -1.0106 | 8.746848 | -6.06673 | 3.15E-07 | 2.90E-06 |
| PRSS35 | -1.01007 | 8.451682 | -8.06018 | 4.51E-10 | 2.18E-08 |
| STK38L | -1.00957 | 9.230729 | -8.33837 | 1.85E-10 | 1.24E-08 |
| FAM129A | -1.00936 | 11.70853 | -5.73357 | 9.50E-07 | 7.28E-06 |
| SYNC | -1.00905 | 8.853427 | -5.35374 | 3.32E-06 | 2.13E-05 |
| CREG1 | 1.007823 | 12.31943 | 7.167124 | 8.24E-09 | 1.62E-07 |
| ADGRF5 | 1.007154 | 10.25004 | 4.125503 | 0.00017 | 0.00067 |
| UBASH3B | 1.005463 | 8.866235 | 8.259577 | 2.38E-10 | 1.44E-08 |
| PRKCDBP | -1.00508 | 11.70779 | -7.34003 | 4.67E-09 | 1.07E-07 |
| CDO1 | -1.00497 | 8.802761 | -8.40594 | 1.49E-10 | 1.10E-08 |
| CEMIP | 1.004951 | 11.12738 | 4.105373 | 0.000181 | 0.000708 |
| PRELP | -1.00333 | 9.050767 | -7.82553 | 9.61E-10 | 3.69E-08 |
| CYTIP | 1.003307 | 9.018555 | 7.138281 | 9.06E-09 | 1.72E-07 |
| DAB2 | 1.001393 | 12.04086 | 6.061412 | 3.20E-07 | 2.94E-06 |
