# Supplementary material for: Differential Gene Expression and Immune Cell Infiltration in Carotid Intraplaque Hemorrhage Identified Using Integrated Bioinformatics Analysis
Source: Front Cardiovasc Med. 2022 May 17;9:818585. doi: 10.3389/fcvm.2022.818585 (PMC9152291; doi:10.3389/fcvm.2022.818585)
Supplement: Supplementary file 4 [file Image_1.pdf]

Supplementary Material

1 Supplementary Figure 1

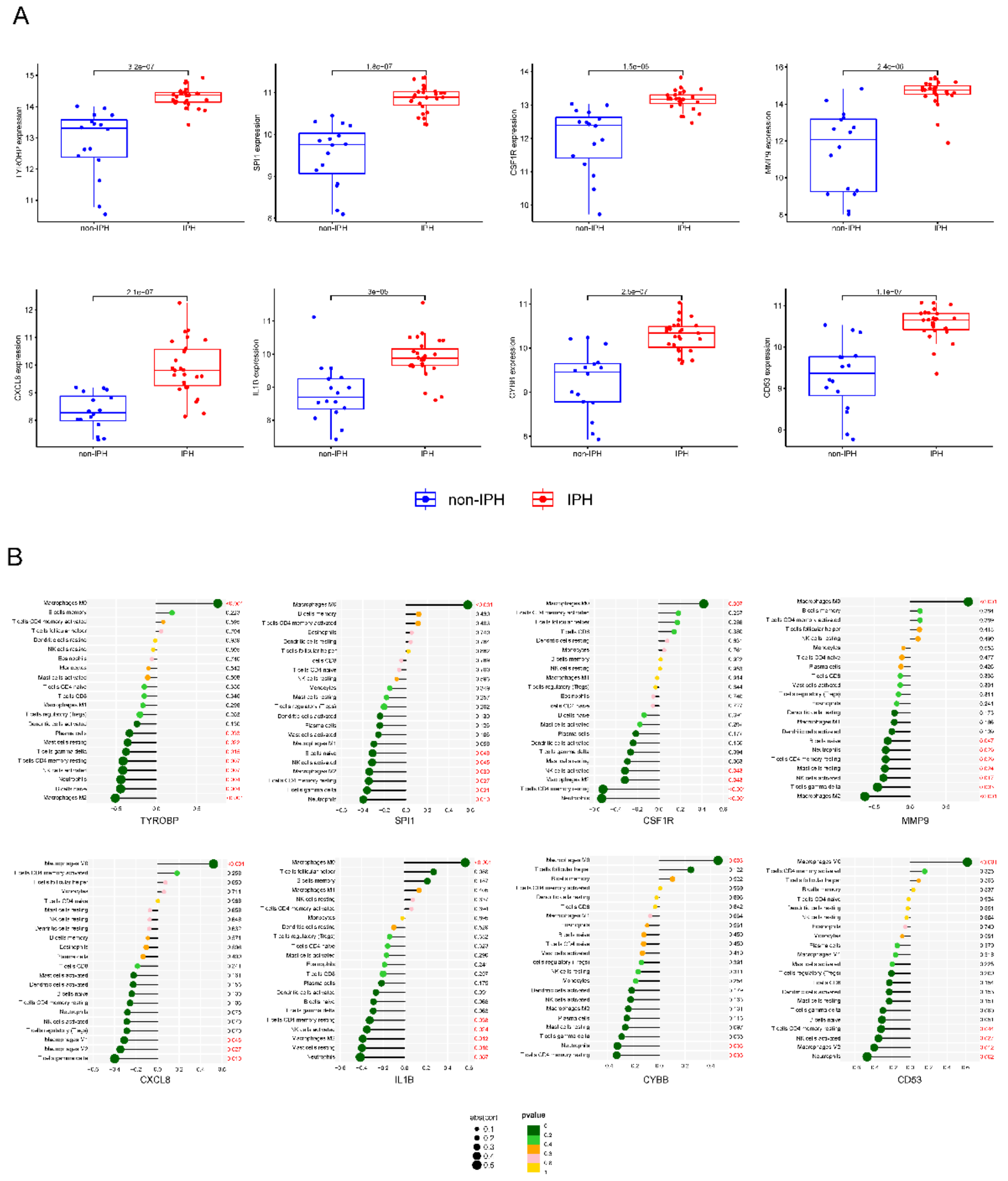

Supplementary Figure 1 | (A) The expression level of 8 genes between IPH and non-IPH samples in dataset GSE163154. **(B)** Correlation between 8 genes and infiltrating immune cells. The size of the dots represents the strength of the correlation between genes and immune cells, and the color of the dots represents the *p*-value. $p < 0.05$ was considered statistically significant.
